# Epidural Needle Guidance Using Viscoelastic Tissue Response

**DOI:** 10.1109/JTEHM.2022.3152391

**Published:** 2022-02-16

**Authors:** Benjamin Scott Simpson, Michael Burns, Robert P. Dick, Leif Saager

**Affiliations:** Department of Electrical Engineering and Computer ScienceUniversity of Michigan 1259 Ann Arbor MI 48109 USA; L3Harris Technologies Salt Lake City UT 84116 USA; Department of AnesthesiologyUniversity of Michigan Medical School 12266 Ann Arbor MI 48109 USA; Department of AnesthesiologyGeorg August University of Göttingen 37075 Göttingen Germany

**Keywords:** Epidural, needle placement, viscoelastic response, machine learning, biomedical engineering

## Abstract

*Objective:* We designed, prototyped, and tested a system that measures the viscoelastic response of tissue using nondestructive mechanical probing, with the goal of aiding clinical providers during epidural needle placement. This system is meant to alert clinicians when an epidural needle is about to strike bone during insertion. *Methods:* During needle insertion, the system periodically mechanically stimulates and collects viscoelastic response information data from the tissue at the needle’s tip using an intra-needle probe. A machine-learning algorithm detects when the needle is close to bone using the series of observed stimulations. *Results:* Tests run on *ex vivo* pig spine show that the system can reliably determine if the needle is pointed at and within 3 mm of bone. *Conclusion:* Our technique can successfully differentiate materials at and in front of the needle’s tip. However, it does not provide the 5 mm of forewarning that we believe would be necessary for use in clinical epidural needle placement. The technique may be of use in other applications requiring tissue differentiation during needle placement or in the intended application with further technical advances. *Clinical and Translational Impact Statement:* This Early/Pre-Clinical Research evaluates the feasibility of a method for helping clinical providers receive feedback during epidural needle insertion—thereby reducing complication rates—without significant alterations from current workflow.

## I. Introduction

**FIGURE 1.**
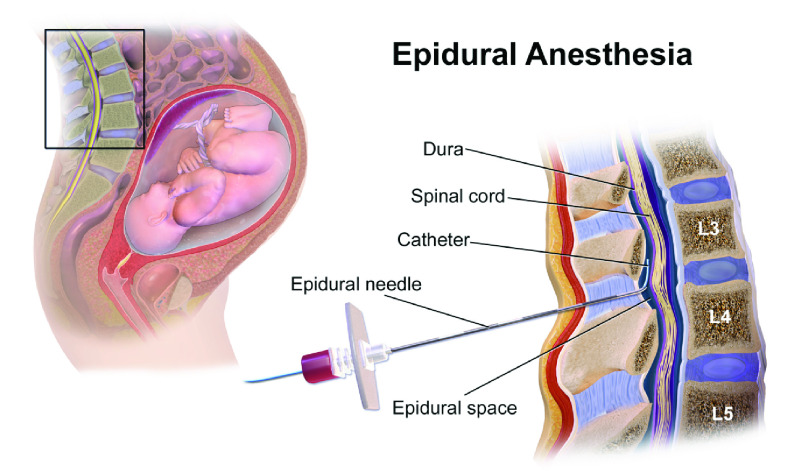
Needle insertion for epidural anesthesia [3].

Several medical procedures require primary physicians or clinicians to blindly insert needles into precise locations with little feedback, leading to high complication rates. One such procedure is epidural needle placement. This procedure is typically performed by anesthesiologists, who must precisely guide a needle between a patient’s vertebrae and into a 2–7 mm wide area known as the epidural space (see Figure 1) [1]. This procedure has a complication rate between 2% and 20% with complications arising from a variety of sources [2]. Improving the efficacy of epidural needle insertion could immediately improve healthcare outcomes, specifically for women undergoing childbirth, people with chronic pain, and patients undergoing surgical procedures aided by epidural placement such as anesthesia, which is used in several types of orthopedic and gastrointestinal surgeries. Other medical procedures requiring precise needle placement may also benefit from such improvements.

A common complication is striking vertebral bone during insertion. Normally this requires the clinician to retract the needle and steer it down a different path, which increases patient discomfort and procedure time. Our goal is to eliminate this complication. We aim to develop a technique to detect an imminent bone strike before it occurs, allowing the needle to be preemptively steered and the strike prevented. Based on the clinical experience of the anesthesiologists on the team, we estimate that 5 mm of forewarning is needed to steer the needle away from bone without first retracting it.

Multiple technologies are in use or are being studied to aid in epidural needle insertion, each with benefits and drawbacks. The most common is ultrasound, which provides visualization of the path to the epidural space and is readily available in hospitals. Additional training is required to interpret ultrasound, and its use adds complexity to a typically unaided procedure. Assistive technologies to reduce the burden of using ultrasound [4]–[8] often require expensive specialized devices. X-ray fluoroscopy is an established assistive technology but requires access to expensive equipment and exposes the patient to ionizing radiation, making it inappropriate to use for labor epidurals. Fiber optic techniques have been shown to differentiate tissue at the needle’s tip, but their range is limited to approximately 2 mm: —too short-ranged to aid in bone strike avoidance [1], [9], [10].

We investigate measuring tissue mechanical properties to aid in epidural needle insertion. This involves applying a mechanical stimulus to tissue and observing the resulting force and motion responses, which vary widely between biological materials. A relevant example is the elastic moduli of bone and soft tissue, which differ by orders of magnitude [11], [12]. Viscoelastic models, which treat a material as having properties of both a fluid (viscosity) and a solid (elasticity), have been found useful in analyzing biological tissue [13]. Viscoelastic response can be used to measure the mechanical response of not just a single tissue but of tissue systems as well. Researchers have shown that viscoelastic measurements of soft tissue over bone vary depending on the thickness of the soft tissue [14]. This is promising for our goal of making a device to alert clinicians when the remaining soft tissue between needle and bone is less than a specified thickness.

Viscoelastic response is frequency dependent. Traditional measurement techniques can involve running multiple stress-relaxation tests or repeatedly subjecting the material to vibrations at different frequencies. Such laboratory measurement techniques are too slow for taking *in vivo* clinical measurements. Fortunately, Zhang [13] has developed a technique for rapid viscoelastic measurements. By applying a mechanical step input to a material and measuring the resulting stress and strain, an impulse response is obtained, which can be converted to a transfer function via a Fourier transform. According to signal processing theory, the transfer function can be used to fully characterize the tissue’s response to arbitrary mechanical strains, thus yielding a fully characterized complex modulus.

In this paper, we design and evaluate a prototype device for avoiding bone strikes during epidural needle insertion. The device measures the viscoelastic properties of the tissue before a needle’s tip with the objective of providing 5 mm of forewarning. Inspired by Zhang’s method [13], viscoelastic data are obtained via a stiff metal probe that replaces the stylet that normally occupies the needle shaft during insertion. Sensors and an actuator are attached to the rear of the probe to produce a mechanical step input and collect the resulting response. Signal processing and machine learning are used to infer the needle’s proximity to bone.

While the device in this paper is table-mounted, a handheld design is envisioned for clinical use. It would attach to the back of the epidural needle during insertion. After the needle is in place, it would be removed so that a catheter could be placed. Unlike other assistive technologies, such a device would be designed to minimally change a clinician’s normal procedure for epidural placement. The machine classifier decision would provide easily interpretable feedback. In contrast to ultrasound, it would not require extensive additional training to interpret or the assistance of an additional technician during procedures. We also believe that such a device, which would require simple disposable probes and modest sensors and computing, would be more cost effective than assistive technologies requiring intensive sensors and processing for visualization or which utilize more expensive consumables, such as specialized through-needle ultrasonic transducers.

This paper makes the following contributions:

1) the design of a device to measure viscoelastic response through a needle and
2) the design and evaluation of a machine-learning technique for determining when a needle is about to strike bone based on viscoelastic measurements.

We evaluate the prototype and analysis techniques on pig spines.

## II. Related Work

### A. Viscoelastic Response

There are multiple methods for measuring viscoelastic response. Stress-relaxation tests measure stress as a material is put under strain and released from that strain, resulting in a hysteresis curve. These data are fit to a mechanical model consisting of a combination of ideal springs (for elasticity) and dashpots (for viscosity) in order to determine the viscoelastic properties. For unknown materials, this requires curve fitting to various mechanical models to find the closest match, thus making it impractical in general practice. Dynamic measurement tests perform stress and strain measurements while sequentially exciting the probe or tissue with a variety of sinusoidal waveforms. Each sinusoidal frequency results in one data point, so multiple tests are needed to obtain the full viscoelastic response, which may prevent use in low-latency, real-time applications [13]. Measurements can also be taken at multiple frequencies by using a chirped frequency signal as input [15].

Zhang [13] proposes a method to obtain the entire viscoelastic response using a single step function as input. This approach treats the tissue as a linear system with strain from a probe as the input and measured stress as the output. By applying a step input and measuring the response, a Fourier domain transfer function can be derived, which can be used to describe the system response to any input, thus fully characterizing the tissue’s viscoelastic response. Simulations show this technique’s results match theoretically derived solutions for both linear and non-linear tissue models, even with non-idealities taken into account such as the non-instantaneous transitions of real-world step function approximations. Experimental results show that the resulting technique successfully differentiates mouse bone tissue in different stages of development [16]. We use the step-function measurement method due to its ability to characterize tissue in real time and the simplicity of implementing a mechanical step stimulus compared to sinusoidal signals.

We cannot implement Zhang’s method exactly. It uses stress and strain measurements while our system measures the force on and position of our mechanical probe, as described in Section III-A. While it is possible to convert from force to stress by taking into account probe cross-sectional area, we cannot measure mechanical strain because its calculation relies upon the total length of the material in question. In this case, that would be the distance from bone, which we are trying to estimate. Instead, we measure the displacement of our mechanical probe. Displacement is commonly used in second-order mechanical system models, which—like viscoelastic models—are also used for calculating time-varying responses to mechanical inputs. Therefore, while our measurement techniques are inspired by Zhang, they cannot be directly compared to his. Despite these differences, we still find that our viscoelastic data are able to differentiate between materials with different viscoelastic properties and be used in bone detection.

Normally viscoelastic measurements are taken *ex vivo*, but our goal is clinical use on patients. We take advantage of the fact that epidurals use large needles designed to accept catheters. Normally, the inside of the needle is sealed using a stylet during insertion, which we replace with a stiff rod for probing tissue. This makes our technique similar to TeMPeST 1-D, a probe that can be inserted through a 12 mm cannula for measuring viscoelastic tissue properties *in vivo* [17]. This device, however, is designed for use with laproscopic equipment and is too large for an epidural needle. To the best of our knowledge, there is no previous work using viscoelastic tissue response to aid in epidural needle placement.

### B. Other Needle Guidance Technologies

Most epidural procedures are performed without assistive technology. The success of these procedures relies on a combination of clinician experience, patient physiology, and patient positioning. Nevertheless, many assistive technologies have been studied and developed, and some see occasional use. The most common of these is ultrasound.

Clinical providers use a standard ultrasound probe to visualize the route the needle should take between vertebrae to the epidural space. This can be done before insertion—a pre-scan—or it can be done during insertion for real-time feedback. The latter generally requires adding another operator to the nominally single-operator procedure. Ultrasound is feasible for lumbar and cervical epidurals, but occlusion by the vertebrae precludes its use in most thoracic epidural procedures [18].

Multiple systems have been studied or developed to simplify ultrasound for epidurals including systems that physically align the needle with the probe [4], [5] and ones that use additional sensors to display the projected needle path on the ultrasound monitor [6], [7]. One group [8] has studied the possibility of placing an ultrasound probe through the measure tissue properties at the needle’s tip. While these systems may obviate the need for a second operator, most of them still require the clinician to be trained to interpret ultrasound data. Our system uses a machine learning algorithm to provide the distance to bone, which is easy to interpret. Also, ultrasound techniques require access to an ultrasound machine, which–while common in hospitals–are complex and expensive. Techniques requiring custom ultrasound probes add more expense. In contrast, our system uses relatively simple hardware and inexpensive metal rods as probes, which we believe will result in lower costs.

X-ray fluoroscopy is an established assistive technology used for epidurals, most commonly in treating chronic pain [9]. This method requires a fluoroscopy room and is impractical in most applications. Furthermore, it exposes the patient to ionizing radiation and is therefore not used in labor epidurals to avoid harming the fetus. Our technique does not require access to a dedicated room and does not expose the patient to ionizing radiation.

Multiple optical techniques have been studied for use in epidural needle insertion. Optical coherence tomography (OCT) uses the timing delay and magnitude of reflected light to measure tissue composition and is analogous to the use of sound waves in B-mode ultrasound [9]. Tang *et al.* have shown its ability to differentiate between tissues during epidural needle insertion [1]. The penetration depth of OCT is approximately 2 mm [9]. Optical reflectance spectroscopy (ORS) measures the reflectance of tissue at the needle’s tip, which can be used to reliably differentiate tissues [9], [10]. Lin *et al.* developed a machine learning method for differentiating between the epidural space and the ligamentum flavum during epidural needle insertion. Their intelligent recognition system uses linear discriminant analysis to classify ORS spectra of tissue at the needle’s tip [10]. These optical techniques are not well suited to our problem, as because they are too short-ranged for steering around bone.

## III. Data Collection Methods

**FIGURE 2.**
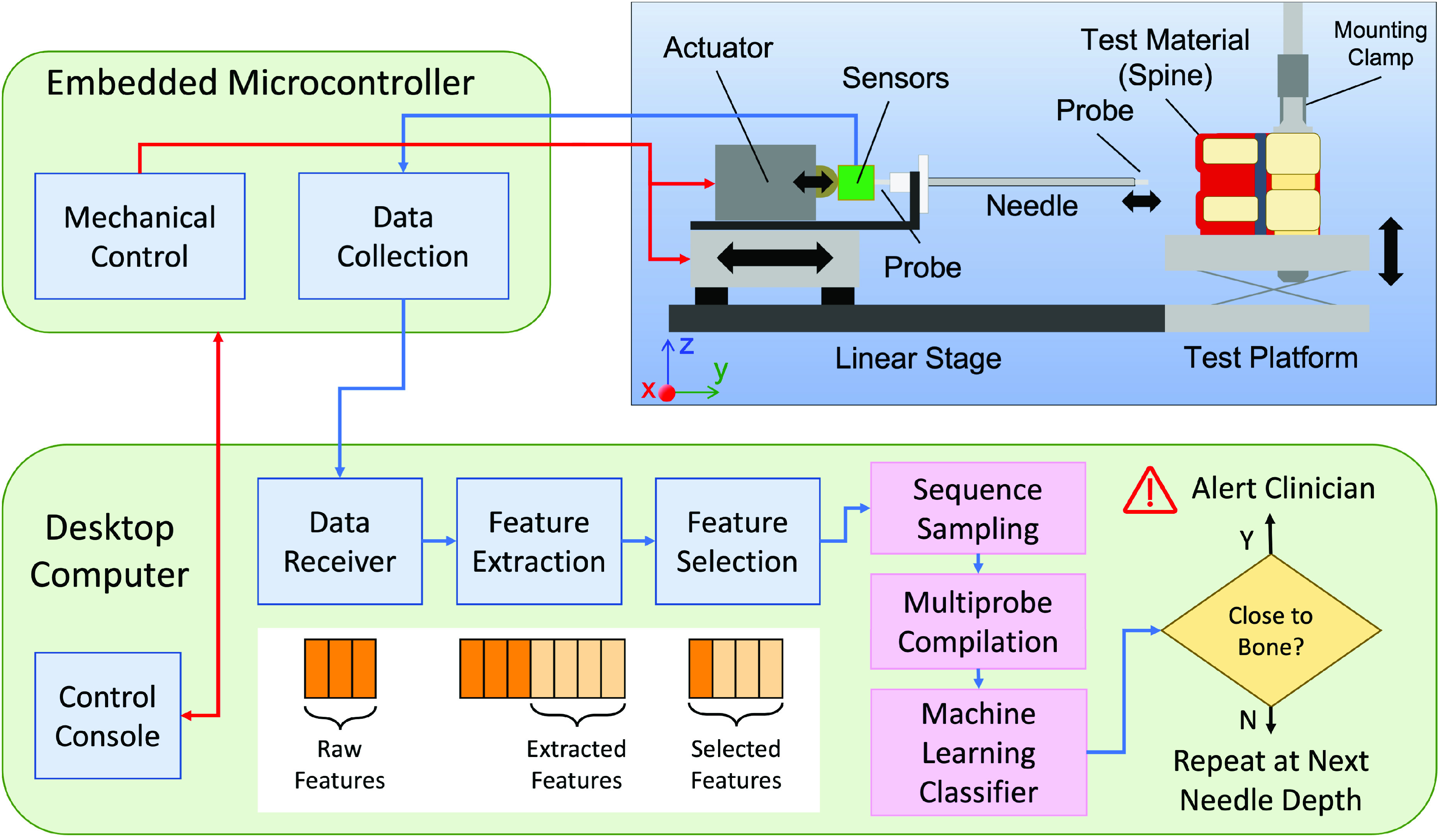
System Test bed block diagram.

In order to develop and evaluate our needle guidance technique, we designed and built a data collection test bed. Figure 2 diagrams its mechanical, computational, and algorithmic features. We envision a final, working version of our needle guidance technique embodied in a handheld device to facilitate clinical use on live patients. However, our test bed uses a computer-driven, bench-mounted linear stage to insert a needle into secured sample material. This design helps us obtain accurate data for our machine learning algorithm. An embedded microcontroller controls the testbed, collects data from its various sensors, and sends data to a desktop computer for processing. Our algorithm consists of preprocessing steps and a machine learning classifier, which infers whether the needle is close to bone. The rest of this section describes the test bed in detail.

### A. Electro-Mechanical Design

The testbed consists of a linear stage that inserts the needle into the test material and an adjustable test platform to hold the test material. A mounting clamp and multiple other clamps and braces (only some of which are shown in Figure 2) secure and immobilize the test material.

Force is applied to the tissue via a stiff stainless steel rod which serves as a probe. The probe is attached to the piston of a solenoid (ROB-10391; SparkFun Electronics; Niwot, CO, USA), with the motion resulting from activating the solenoid approximating a step function. The probe is passed through the needle and cut to length so that when the solenoid is not actuated, the probe is completely within the needle, and when the solenoid is actuated, the probe extends beyond the needle by approximately 2 mm. Importantly, the probe is blunt so that it deforms the tissue rather than cutting it.

The needle used (17G $\times3.5$ in, SKU 4076; Cadence Science; Cranston, RI, USA) has a standard beveled tip and differs from the Tuohy needles most commonly used for epidurals in that it has a completely straight shaft. The curve at the distal end a Tuohy needle shaft would not allow the probe to pass through without high friction.

Our testbed uses multiple sensors for detecting the force and distance curves that constitute viscoelastic response. A force sensor (LCM100; FUTEK; Irvine, CA, USA) placed between the probe and solenoid piston directly measures force. It has a resolution of $2.9\times 10^{-2}$ N. A magnetic linear encoder (iC-MU; iC Haus; Bodenheim, Germany) measures the probe movement with a resolution of $3.1\times 10^{-4}$ mm.

We designed and built an embedded system for controlling and collecting data from our testbed. Based around an S7G2 microcontroller (Renesas Electronics Corporation; Tokyo, Japan), it uses custom firmware to advance the stepper motor, actuate the probe, collect and preprocess data from sensors, and transmit those data to a computer for further analysis. Care was taken in the printed circuit board (PCB) design to electrically isolate the power circuits for the actuators from the sensors to reduce measurement noise.

**FIGURE 3.**
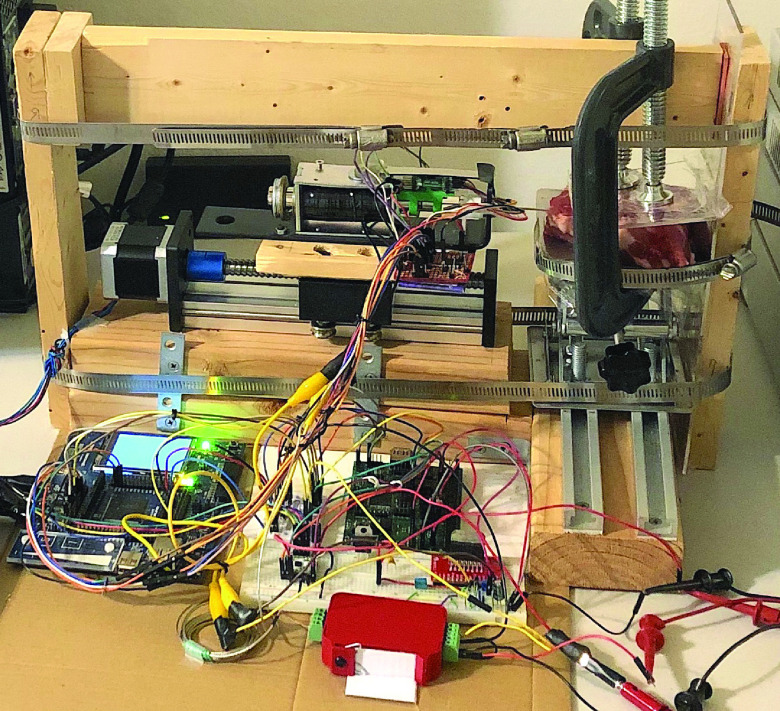
Data collection test bed showing mechanical components, sensor circuitry, embedded microcontroller, clamped sample, and bracing. This photo has been flipped horizontally to match the orientation of the mechanical components in Figure 2.

Our completed testbed is pictured in Figure 3.

### B. Data Collection

A working clinical version of our technique would periodically collect data as a practitioner inserts the epidural needle and would send an alert when the needle is close to bone. In contrast, the goal of our test bed is to obtain labelled data for training and evaluating our classification algorithm. To this end, the test bed mimics an epidural needle insertion resulting in accidental bone strike rather than successful placement.

Securing the tissue so that the needle is aimed at bone, the needle is advanced in increments of 0.5 mm. At each needle depth, the probe is actuated to generate a step input in the tissue, during which the system captures sensor data. Once the sensor data are captured, the probe retracts, and the sensor data and needle depth are recorded as a single probe event. To obtain more data and determine the impact of hysteresis in visco-elastic tissue, the actuation and recording are repeated to capture three probe events at each needle depth. Probe events only occur when the needle is stationary, and no data collection occurs while the needle is advancing.

**FIGURE 4.**
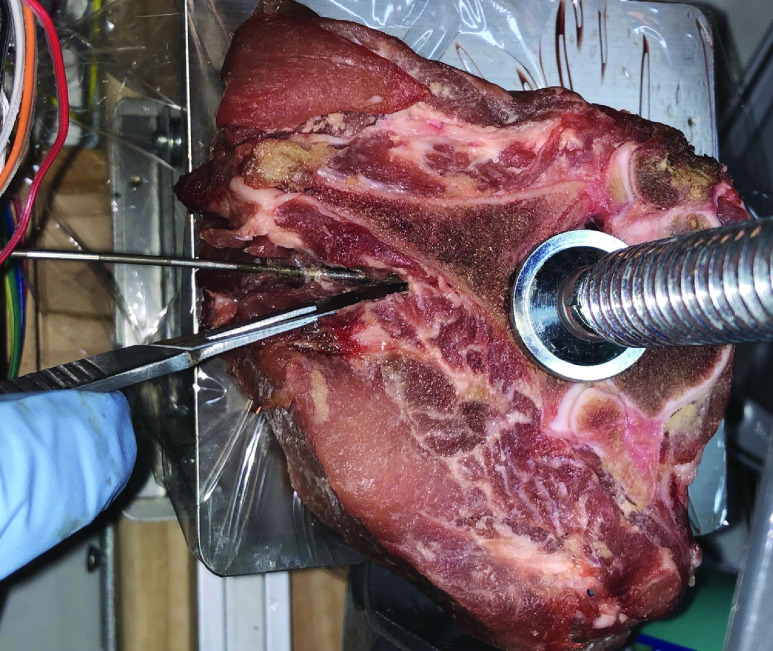
Dissection of test material with needle in place to verify bone strike.

When the needle strikes bone, the test stops and the tissue is dissected to verify the bone strike (see Figure 4). If bone was struck, the final needle depth is recorded as the bone strike depth. All probe events in the test are then labelled by subtracting their recorded depth from the bone strike depth, yielding the distance from bone for each probe event. The sequence of probe events constitutes the data for one needle insertion, which is the basic input to our classification algorithm. For additional information on detecting bone strikes, see Appendix III.

Each probe event lasts 0.25 s and samples 100 data points, yielding a sampling rate of 400 Hz. According to sampling theory, this allows us to measure signals up to 200 Hz, which covers many frequency ranges found useful in past work [14]. Both the force and probe position (also referred to as distance in this manuscript) data are sampled, yielding two time series of 100 points each as the raw data for each probe event.

**FIGURE 5.**
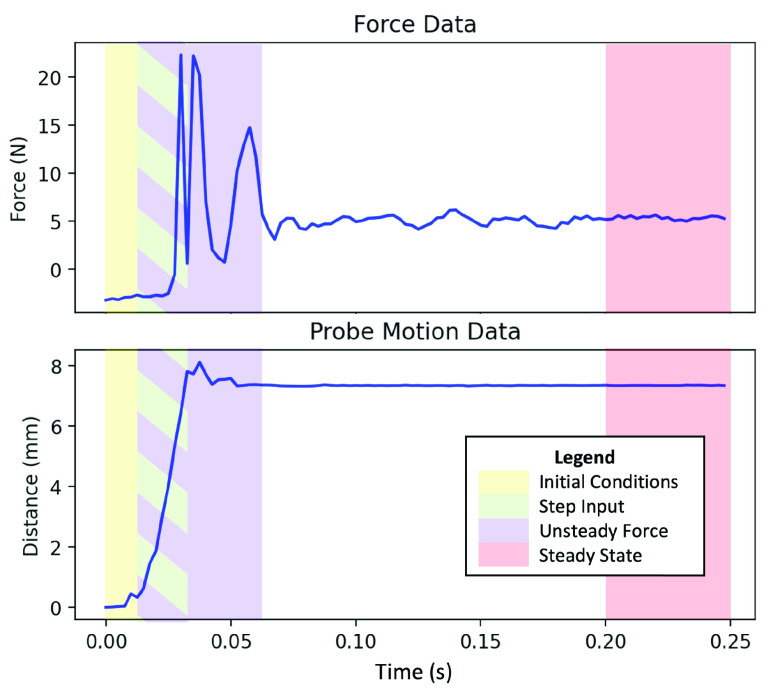
Example of raw force and distance data from a single probe event with the subsequences used in feature extraction highlighted. These subsequences are initial conditions (0–12.5 ms; yellow), step input (12.5–30 ms; green), unsteady force (12.5–60 ms; purple), and steady state (200–250 ms; red). The subsequence timing windows are equivalent the same for all probe events.

Figure 5 shows an example of the raw force and distance data from a single probe event. Note that the probe motion does not constitute a perfect step input. Though it has a mechanical hard end stop, it is impossible to achieve instantaneous probe motion in a real system. If we were using this to calculate the system’s transfer function, the approximately 18 ms travel time of the probe would translate to an error ratio greater than 3 dB at frequencies above 25 Hz. However, because we use both the probe position (input) and force (output), our primary interest is in the relationship between them rather than the transfer function. Thus the 25 Hz corner frequency on our error is a pessimistic lower bound on the maximum frequency our technique can measure.

## IV. Preliminary Experiments

We ran several preliminary experiments to determine whether our testbed was able ability to collect useful viscoelastic data and to verify a hypothesis that is helpful when using mechanical viscoelastic response for needle guidance.

### A. Distinguishing Emulated Adipose and Ligament Tissue

We aim to establish that the proposed technique is capable of distinguishing between viscoelastic materials of different hardnesses by measuring their responses when mechanically stimulated using step function approximations. To this end, we prepared hard and soft silicone samples. Using silicone simplifies the classification problem: it can be made to different hardnesses and is more spatially homogeneous, temporally stable, and easier to work with than biological soft tissue samples. One sample was designed to have the consistency of ligament tissue while the other was designed to have the consistency of adipose tissue. A needle passes through both types of tissue during an epidural insertion. The raw data for probe events taken in the middle of each sample showed markedly different characteristics. While the distance data only showed small differences, there were significantly higher force readings in the ligament-like silicone than in the adipose-like silicone. This proved that the viscoelastic data our device collects can be used to distinguish between tissues of different types. Further details on this experiment, including graphs of the data, can be found in Appendix V. Section VI will explain evaluation on biological tissue.

### B. Directionally Selective Remote Detection of Hard Materials

It is common for a needle to pass close to bone without striking it during a correct epidural needle insertion. Therefore, our goal is to detect nearby hard material in the needle path, without detecting off-axis hard material. Were we unable to do this, our system would be inundated with false positives. We hypothesize that the information collected by our needle-axis-aligned directional probing is directional in nature.

To test this hypothesis, we prepared a mock bone-tissue interface consisting of 20A durometer silicone, which has a hardness similar to ligament, juxtaposed with polyether ether ketone (PEEK), which has a hardness similar to bone. To test directionality, we compare probe events taken at the same distance from PEEK but with the needle either pointing at (perpendicular to) or not at (parallel to) the PEEK. We took both parallel and perpendicular measurements with offsets from PEEK ranging from 1 mm to 8 mm in 1 mm increments. For each depth, we trained a linear support vector machine (SVM) binary classifier using 5-fold cross validation to differentiate between parallel measurements and perpendicular measurements. This did not follow the data analysis pipeline in Figure 2 but simply used the raw time-series data from each probe event as input to the SVM. We had more parallel measurements than perpendicular measurements, so to avoid errors due to differently-sized training sets, each SVM was trained with the 30 perpendicular measurements taken at the specified depth and a set of 30 randomly selected parallel measurements taken at the same depth. The SVMs were trained three times using different random selections of parallel data and splitting of testing/training data during cross validation, and the resulting classification accuracies were averaged. Using this methodology, our SVMs correctly classi fied a given probe event as perpendicular or parallel with an accuracy of 99.8%, averaged across all eight distances.

This high degree of separability implies that our measured viscoelastic data are sufficient to determine whether a needle is pointed at bone-like material or is near it but not pointed at it. These results, combined with our testing on silicones of different hardnesses, indicate that our device is able to classify based on material properties and the needle’s orientation to interfaces between materials with differing properties.

## V. Classification Algorithm

Our classification problem presents unique challenges. Its input is an ordered series of probe events, which each contain time series data from two sensors. The output is a binary decision indicating whether the needle is close to bone. Since the goal is to avoid striking bone, we must infer distance to bone using only probe events at or prior to the current needle depth. To achieve this, our classification algorithm examines the probe events of a needle insertion in order, deciding whether the needle is close to bone at each step. The needle depth of the first probe event classified as occurring within 5 mm of bone becomes the detection depth for the entire needle insertion. The data processing and decision making steps used on each probe event are shown in Figure 2 and are described below.

The first steps preprocess the raw data for individual probe events into feature vectors for the machine learning classifier. The feature extraction step generates additional features from the raw force and distance sequences. The mean, standard deviation, skewness, and kurtosis are extracted as individual scalar features. FFTs are also performed on the raw sequences and added as vector features. Note that force and distance are treated as two separate sequences for feature extraction. We also found it useful to make the same set of statistical calculations and perform FFTs on subsequences of the probe data. These subsequences were manually selected based on observations of the raw data. They constitute regions of interest in the probe event such as the period of time when the probe is moving or the period when the force reading has reached steady state. The exact subsequences can be seen in Figure 5. After feature extraction, the feature selection step removes any extraneous features, such as the raw force or distance data, that are not used by the current feature set. After this, the features are concatenated to form the feature vector. In our experiments, we test multiple feature sets, which are described in Section VI.

Sequence sampling randomly samples one of the three probe events collected by our test bed at each depth of a needle insertion to create needle insertion sequences with only one probe event at each depth. Multiprobe compilation concatenates the feature vector at the current depth with the feature vectors at the $N - 1$ previous depths. This allows the machine learning classifier to make a decision based on a window of $N$ probe events, thus making use of the sequential nature of the needle insertion data rather than making decisions based solely on the current probe event.

The machine learning classifier takes the fully processed feature vector as input and outputs a binary classifier decision indicating whether the needle is close to bone. Any binary classifier might be used for this purpose. We experimentally evaluated the performance of multiple classifiers under multiple training regimes.

To evaluate the system’s accuracy, the depth of the first probe event that is classified as close to bone is recorded and compared to the ground truth close-to-bone depth for that needle insertion, which we have set as 5 mm before bone strike. This yields an error value in millimeters for each test. A negative error means the system’s classification distance was earlier (further from bone) than our 5 mm threshold while a positive error indicates the classification distance occurred later (closer to bone) than the threshold.

## VI. Experiments

**FIGURE 6.**
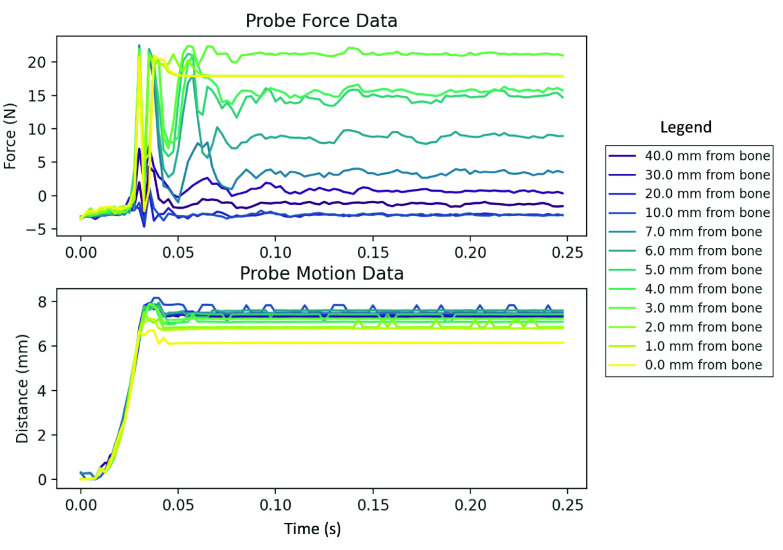
Force and distance data from a single insertion. For clarity only some a subset of probe events are shown, with more probes that are close to bone being shown as they show more change more between depths as compared to than probe s events that are far from bone. Figures 9 and 10 in Appendix I contain graphs showing all the probe events for this same needle insertion.

We experimentally measured 20 needle insertions using 3–5 cm thick slices of pig spine as our test material, which we obtained from a local butcher. Slices were purchased at different times, making it very unlikely that they all came from the same pig. We generally limited ourselves to doing two needle insertions on each slice—one on each side of the spinous process—to avoid having needle insertions in close proximity to each other. Bone strike depths ranged from 17.5 mm to 54 mm with an average depth of 40.5 mm and a median depth of 43.5 mm. The tissue temperature during testing had a range of 20.4–22.3 °C. Figure 6 shows raw probe event data from one of the needle insertions.

We evaluated different feature sets both by inspecting feature values for visible changes near or before our desired classification depth and by running our classification algorithm on different groups of features. We ultimately selected four feature sets to show in this manuscript. The Force FFT $\cup $ Dist FFT feature set consists of the FFTs of the force and distance data; this is inspired by Zhang’s method [13], which involves uses the ratio of the stress FFT divided by the strain FFT. Instead of finding a ratio between our force and distance viscoelastic data, we concatenate the two in order to retain more complete data for the classifiers. The Force $\cup $ Dist feature set simply uses the raw, time-domain force and distance sensor data. The Raw $\cup $ Stats feature set contains the raw force and distance sensor data as well as mean and/or standard deviation values for certain subsequences which were found to perform well in preliminary testing. The Subsequence Force FFTs feature set consists of FFTs of the force data contained in the subsequences in Figure 5; only force data are used as the distance subsequence FFT data were found to be less helpful. The specific features used in each feature set and details on the information provided by individual features are described in Appendix VII.

We selected three machine learning classifiers for our experiments. The first, SVM, was selected for its simplicity and versatility. We use a linear kernel since others did not generally improve accuracy. Random Forest was the second classifier. Unlike SVM, which searches for an optimal hyperplane to separate the data classes, Random Forest relies on results from multiple decision trees to classify data. Our third classifier was eXtreme Gradient Boosting (XGBoost) [19]. Like Random Forest, XGBoost relies on an ensemble of decision trees; however, rather than using random feature and data splits to generate multiple trees, XGBoost uses a technique known as gradient boosting to generate the classifier forest so that each new decision tree reduces the decision error of the ensemble of previously generated trees [20].

For our experiments, SVM and Random Forest are trained using binary labels that divide all probe events into two categories: “within 5 mm of bone” and “not within 5 mm of bone.” These are calculated from the regular distance-from-bone labels of each probe event by applying a binary threshold. Probe events within 5 mm of bone receive a label of ‘1’ while those not within 5 mm of bone receive a label of ‘0’. Their For the SVM and Random Forest classifiers, the output for each probe event is a binary decision on the category to which the probe event belongs. XGBoost uses the standard distance-from-bone labels and outputs an estimated distance from bone for each probe event. The estimated distance is thresholded using our classification depth of 5 mm to reach a binary decision.

**FIGURE 7.**
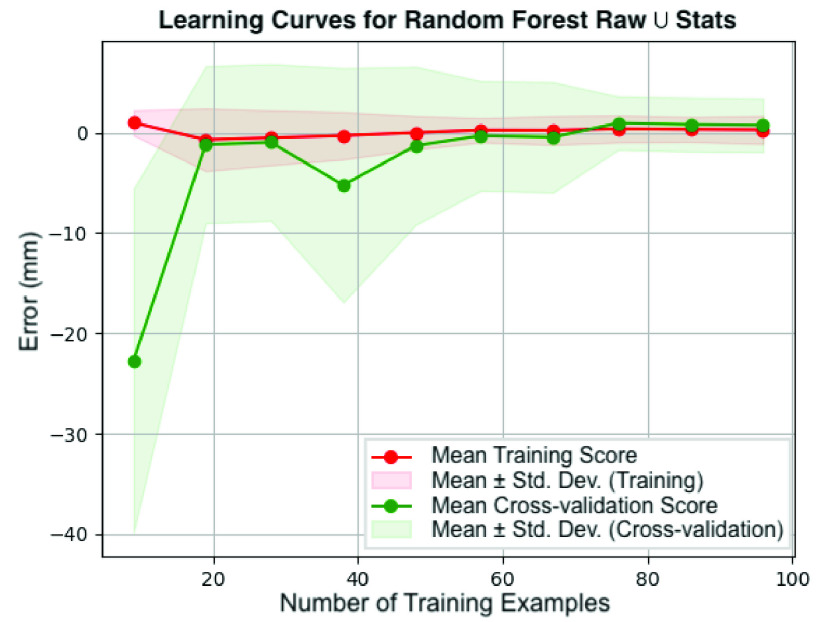
Learning curve showing results for a Random Forest classifier using the Raw $\cup $ Stats feature set with a multiprobe number of 3 and 5-fold cross-validation as the number of sequences sampled is swept. Ten sequences were extracted per needle insertion from the training set, resulting in 120 total sequences. Since this learning curve uses 5-fold cross-validation, 80% of the total sequences are used for training and 20% for testing; thus, the number of training examples on the x-axis range from 9 to 96. For each set of results, the lines indicate the mean error while the similarly colored shaded regions around each line show ±1 standard deviation away from the mean error. Convergence between the two sets can be seen at around 80 training samples.

To determine the values for the number of sequences to sample and the number of probe s events to concatenate, we ran sweeps of those two variables across all machine learning classifiers and multiple feature sets. We trained these classifiers using both a train/validation split as well as cross-validation on the training set. Figure 7 shows an example of the results of a sweep across the number of sequences for a fixed classifier, feature set, and multiprobe number. We select the final number of sequences per needle insertion to extract from values that show low training and validation errors.

In our experiments, we use two training regimes for our classifiers. The first is a test/train split where twelve needle insertions are used to train the machine learning classifiers and four are used to test it them. The other four needle insertions formed a validation set used as a test set during algorithm development. The other training regime is referred to as leave-one-group-out or LOGO. This regime trains the classifier on eleven of the needle insertions in the training set and evaluates the algorithm with the remaining needle insertion. This process is repeated with a different needle insertion reserved for evaluation until each of the twelve insertions have served as the evaluation set.

We evaluate our experiments using three error measures calculated from the set of individual needle insertion errors: average error, mean absolute error (MAE), and root mean square error (RMSE). Average error indicates whether the algorithm is classifying the needle as near bone too early or too late. MAE is useful for determining how far away from the threshold point classifications are made. RMSE emphasizes more extreme errors.

## VII. Results

Table 1 shows the results of our experiments. Multiple general trends are apparent. One trend is that the test/train split training regime usually obtains smaller errors than LOGO. However, there are a few notable cases—which are discussed below—where LOGO does better in certain error measures. Another notable trend is that among the results that achieve low overall error rates, all of them have a positive average error, indicating that they tend to classify the needle as close to bone late, i.e., after it has already passed the 5 mm threshold. We discuss this in-depth in Section VIII.

**TABLE 1:** Experimental Results:. See Section V for a Description of Multiprobe Compilation and Section VI for Descriptions of the Other Columns. Highlighted Rows Indicate Specific Results Discussed in the Text. Identification and Analysis of Trends Can be Found in Section VII and Section VIII, Respectively

Algorithm	Feature Set	Multiprobe Compilation (N)	Sequences Extracted per Needle Insertion	Training Regime	Avg Error (mm)	MAE (mm)	RMSE (mm)
Random Forest	Force FFT $\cup $ Dist FFT	3	10	test/train	1.80	1.80	1.87
Random Forest	Force FFT $\cup $ Dist FFT	3	10	LOGO	−2.14	4.30	7.02
Random Forest	Force $\cup $ Dist	3	10	test/train	2.20	2.20	2.33
Random Forest	Force $\cup $ Dist	3	10	LOGO	0.81	2.64	3.01
Random Forest	Raw $\cup $ Stats	3	10	test/train	2.08	2.08	2.29
Random Forest	Raw $\cup $ Stats	3	10	LOGO	0.53	2.76	3.19
Random Forest	Subsequence Force FFTs	3	10	test/train	1.73	1.73	1.83
Random Forest	Subsequence Force FFTs	3	10	LOGO	−2.28	4.60	7.61
SVM	Force FFT $\cup $ Dist FFT	5	12	test/train	−10.43	11.01	16.23
SVM	Force FFT $\cup $ Dist FFT	5	12	LOGO	−16.42	17.02	21.56
SVM	Force $\cup $ Dist	5	25	test/train	−3.34	5.09	9.15
SVM	Force $\cup $ Dist	5	25	LOGO	−10.77	12.22	18.60
SVM	Raw $\cup $ Stats	5	12	test/train	−4.53	6.07	9.65
SVM	Raw $\cup $ Stats	5	12	LOGO	−10.13	11.59	17.89
SVM	Subsequence Force FFTs	5	25	test/train	1.67	1.67	1.85
SVM	Subsequence Force FFTs	5	25	LOGO	−2.88	5.93	10.09
XGBoost	Force FFT $\cup $ Dist FFT	3	10	test/train	2.09	2.16	2.24
XGBoost	Force FFT $\cup $ Dist FFT	5	10	test/train	2.13	2.13	2.26
XGBoost	Force $\cup $ Dist	3	10	test/train	2.29	2.29	2.38
XGBoost	Force $\cup $ Dist	5	10	test/train	1.35	2.95	3.49
XGBoost	Raw $\cup $ Stats	3	10	test/train	2.29	2.29	2.38
XGBoost	Raw $\cup $ Stats	5	10	test/train	2.44	2.44	2.56
XGBoost	Subsequence Force FFTs	3	10	test/train	1.85	2.65	3.54
XGBoost	Subsequence Force FFTs	5	10	test/train	2.48	2.48	2.66

An interesting trend becomes apparent when one looks at the results that achieve the lowest error rates in specific categories. The best results for average error were under 1 mm and were achieved by Random Forest using the Force $\cup $ Dist and Raw $\cup $ Stats feature sets and the LOGO training regime; however, MAE and RMSE were 2 mm or more for these cases, meaning that these cases had low bias but relatively high variance.

The best results for MAE and RMSE occur in the same cases and were obtained by SVM using the Subsequence Force FFTs feature set and by Random Forest using the Subsequence Force FFTs and Force FFT $\cup $ Dist FFT feature sets. All of these cases use the test/train split training regime. These error values range between 1.67 mm and 1.87 mm. The fact that both of these error measures are in such a small range implies that the contribution of rare high-error inferences to aggregate error is minimal. Furthermore, the average error is also within this range, implying that the majority of bone detection classifications were clustered around the same depth in each test. Thus, these cases result in high bias but low variance.

As our best results are split into two groups, we analyze both. The low-bias-high-variance best results (Random Forest, Subsequence Force FFTs, test/train) show that we can generally detect when the needle is within approximately 2–8 mm of bone using our classification label of 5 mm and enforcing an RMSE of less than 3.19 mm. The best high-bias-low-variance results (SVM, Subsequence Force FFTs, test/train) show that we can consistently detect when the needle is within 3.33 mm of bone based on an average error and MAE of 1.67 mm.

These results indicate that, in general, our bone detection method works: it detects when the needle is near bone before the needle strikes the bone 20% the error of random guessing which, during a theoretical 40 mm needle insertion, would result in an average error of −15 mm, MAE of 15.68 mm, and RMSE of 19.02 mm. However, these results do not meet our self-identified standard for this technique to be useful in epidural needle insertion, which we believe would require alerting a clinician when the needle is within 5 mm of bone with a detection error of ±1 mm. The next section further analyzes the results to determine why our results end up in two main groups and how they might be improved.

## VIII. Discussion

Our preliminary experiments showed that our method can differentiate tissues and provide information on the needle’s orientation in relation to a bone-tissue interface. For our technique to work, these classifiable differences in material properties must occur as an epidural needle approaches bone. Other preliminary needle insertion data from sheep and pig spine showed some changes as the needle approached bone, with the steady-state force measurement often increasing. This and previous research that showed differences in measurable viscoelastic tissue properties for thick and thin layers of soft tissue over bone [14] gave us reason to believe that we would be able to determine when the needle is approaching bone.

**FIGURE 8.**
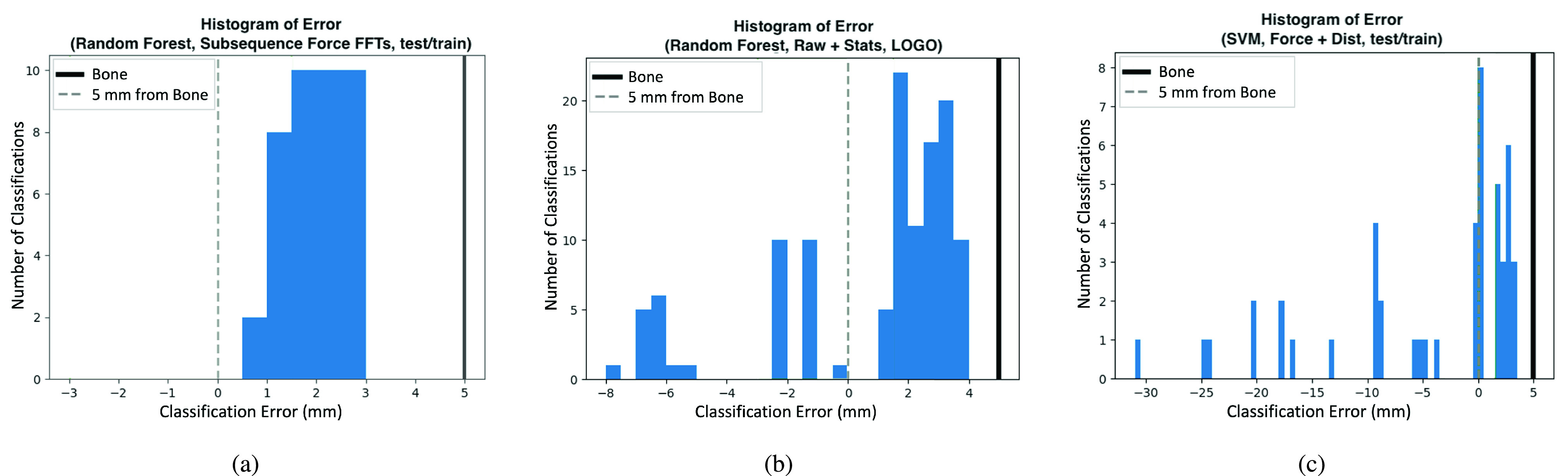
(a) Histogram of a high-bias-low-variance classifier from Table 1 (Random Forest, Subsequence Force FFTs, test/train). (b) Histogram of a low-bias-high-variance classifier from Table 1 (Random Forest, Raw $\cup $ Stats, LOGO). (c) Histogram of a classifier with poor error metric measures from autoref tab:MultiProbeResults (SVM, Force $\cup $ Dist, test/train).

Though our classifiers had either low bias or low variability, none of them achieved both. Further examining the data shows why. Figure 8 shows histograms of bone detection errors on our needle insertion training set for specific classifiers in Table 1. Figure 8(a) shows a classifier with high bias and low variance while Figure 8(b) shows a classifier with low bias and high variance. It can be seen that in both cases, most close-to-bone classifications occur in a cluster with a center approximately in the range of 1.5–2 mm of error or, equivalently, 3–3.5 mm away from bone. The main difference between the low-bias-high-variability and the high-bias-low-variability classifiers is that the latter are more concentrated in the main cluster while the former have outliers which bring the average error closer to 0 mm. This clustering behavior can even be seen in some classifiers that have very high error rates as shown in Figure 8(c): there still exists a main cluster centered at the same location, but there are many early detection outliers that ruin the averages.

These results indicate that our bone detection method works: it detects when the needle is within 3 mm of bone, before the needle strikes bone. However, as currently implemented, it is unable to provide this warning soon enough to be of clinical use in epidural needle insertion. The measurable viscoelastic properties of soft spinal tissue near bone do not change enough at a distance far enough away from bone for our technique to give clinical providers the 5 mm of forewarning that we estimate are needed to steer the needle around bone without first retracting the needle. Our data show that this change does not occur until the needle tip is within 3–3.5 mm of bone or, equivalently, until the extended probe tip is within 1–1.5 mm of bone. This is verified by the clusters shown in the histograms.

This also explains the bias-variance tradeoff seen in our classifiers. The classifiers are being tasked with finding when the needle is within 5 mm of bone, but the features do not appear to meaningfully change until the needle is within 3.5 mm of bone. As a consequence, the inference algorithms are unable to distinguish between these (overlapping) classes until within 3.5 mm of bone, where features begin to diverge.

It may be possible to increase detection range with improvements to our technique. Increasing the distance that the probe extends beyond the needle would allow it to collect data at a greater range from the needle’s tip. However, at some point this would open questions about how far the probe can extend without risking tissue damage. Algorithmic improvements could also help. Our current classification algorithm does not make any underlying assumptions about the material being tested. Incorporating specific tissue models may improve results. While our algorithm uses data from multiple probe events, it is limited to a small window of probe events. Machine learning techniques more specifically designed for sequential data such as recurrent neural networks may be able to find enough information in needle insertion sequences to detect bone sooner. These neural-network-based techniques require a large amount of training data.

Our current viscoelastic response technique is promising for medical applications beyond epidural placement. A key contribution is showing the ability to directionally classify tissue through a needle with non-destructive mechanical probing. With results showing its ability to collect data for tissue classification in a unique through-needle manner, our technique might be used for other blind needle placement medical procedures, such as non-invasive tissue biopsies, laparoscopic instrument placement, and fluid drainage techniques.

We also noticed a practical insight that may be useful I in further developing machine learning models for similar applications. Table 1 shows that models which produced high-bias-low-variance results mostly used the Subsequence Force FFTs and Force FFT $\cup $ Dist FFT feature sets. This implies that FFT-based features may be less susceptible to noise and more useful in extracting the underlying information in the data than time-domain sequences or statistically derived scalars.

## IX. Open Questions

The following questions may merit consideration in further research.

1) Since tissue is viscoelastic, it may be deformed after one or more probe events. Some force graphs of preliminary data showed small differences based on the order of probe events at the same depth, but preliminary classifiers that explicitly took probe ordering into account received no benefit, so we stopped considering probe order. It is possible that classifiers with more advanced features—including sequence sampling, which had not been implemented in these preliminary tests—may be impacted by these small differences and could benefit from taking probe ordering into account.
2) Our directionality test only compares when the needle is pointed perpendicular to or parallel to the simulated tissue-bone interface. Determining the change in measured tissue response as a function of angle might be useful.
3) It remains to be determined how effectively our device can communicate to a practitioner during a real-time insertion. Based on computation time estimates of our algorithms and needle insertion speed estimates calculated from a small set of author-conducted interviews with anesthesiologists and anesthesiologist residents, the computational latency of our system is low enough to allow for real-time use. An evaluation measuring needle insertion speeds and practitioner reaction to feedback would be beneficial.

## X. Conclusion

We developed and evaluated a novel technique for avoiding bone strike during epidural needle insertion by measuring the viscoelastic response of tissue at the tip of the epidural needle. We verify that the technique can successfully differentiate tissue at the needle’s tip and identify when the needle is pointed at and 3 mm away from bone. This does not give us the desired forewarning of 5 mm that we estimate would be necessary for use in clinical epidural needle insertion. The probing technique may be useful in other applications requiring blind needle insertion or *in vivo* tissue classification. Lengthening the probe or further improvement to the classification algorithm might yield a longer detection range.

## Supplementary Materials

Supplementary materials
