# Supplementary material for: Epidural Needle Guidance Using Viscoelastic Tissue Response
Source: IEEE J Transl Eng Health Med. 2022 Feb 16;10:4900611. doi: 10.1109/JTEHM.2022.3152391 (PMC8932520; doi:10.1109/JTEHM.2022.3152391)
Supplement: Supplementary materials [file supp1-3152391.pdf]

APPENDIX I
PROBE DATA AT DIFFERENT DEPTHS

This appendix illustrates how our probe data change with respect to distance from bone and provides some analysis and explanation on the changes seen. Figures 9 and 10 show the distance and force data for each probe event in a test run. Figure 11 is a photo of the sample used in the test run with the sample partially dissected to show the needle path. In the graphs, each trace represents one probe event. The trace colors change from purple to yellow as the needle is inserted deeper. The purple traces for the earliest probes are mostly covered by later blue, green, and yellow traces.

The distance data do not change much until the probe begins striking bone at 2 mm. At that point, the distance the probe travels decreases as it moves toward bone. The distance stops decreasing once the needle has struck bone.

The force data are also similar between probe events with the exception of the steady-state force increasing as the needle passes ligaments and approaches bone. It can be seen from the final probe events in Figure 10 that the force sensor readings saturate. This occurs when the probe begins striking bone. Of our 20 insertions, force sensor saturation starts occurring at 2.5 mm from bone with only one insertion saturated at that depth. At 2.0 mm, two insertions are saturated, and at 1 mm, 16 insertions are saturated. This is consistent with the distance that the probe extended beyond the needle in our tests which ranged from approximately 1.2–2.0 mm . The two probe events that saturated at or before 2 mm still saturated within 1 mm of this distance. The discrepancy may be caused by estimation error on the collision depth, which was based on the probe motion data.

While this saturation data necessarily affected our algorithm's training to identify bone strike, it did not depend wholly on this force sensor saturation as evidenced by its ability to detect bone at a distance greater than 2 mm. While it may have been possible to adjust the force sensor gain settings to avoid measurement saturation, this would not have alleviated the issue of qualitatively different force readings when the probe began to strike bone. Even if the sensor voltage readings did not saturate, the actual force would "saturate" by virtue of the probe striking a hard surface rather than soft tissue. This change is already captured by the distance data. Leaving the force sensor at its current gain settings allows for a more relevant dynamic range and thus better data resolution for measuring force on the regular probe events.

APPENDIX II
ON NEEDLE BENDING

Our tests were not conducted in a manner to specifically avoid or measure frictional force due to needle bending, so we are unsure of its exact effect on our results. However, we are reasonably confident that needle bending did not significantly affect our results.

Our force measurements only measure force on the probe, not on the needle itself. We expect that both stiffer tissue at the needle's tip and increased friction due to probe bending would increase the force measured by the probe. There is no

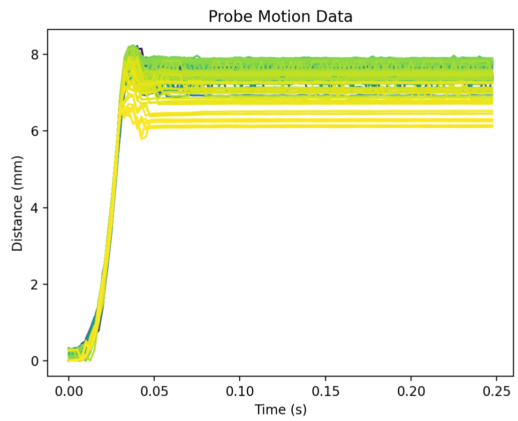

Fig. 9: Distance data for all probe events during a single test.

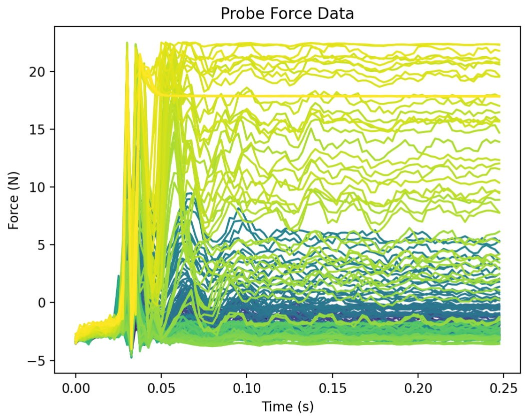

Fig. 10: Force data for all probe events during a single test.

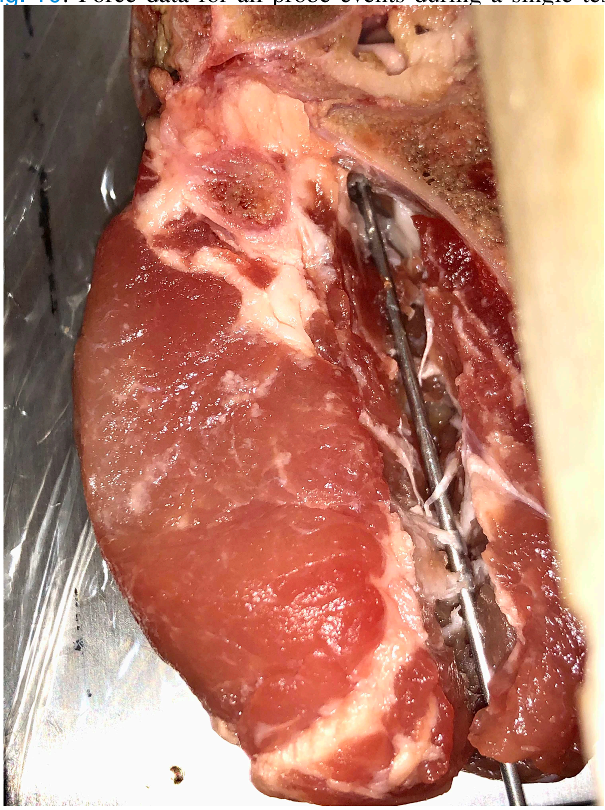

Fig. 11: Post-test dissection to verify bone strike and show the path of the needle through the sample.

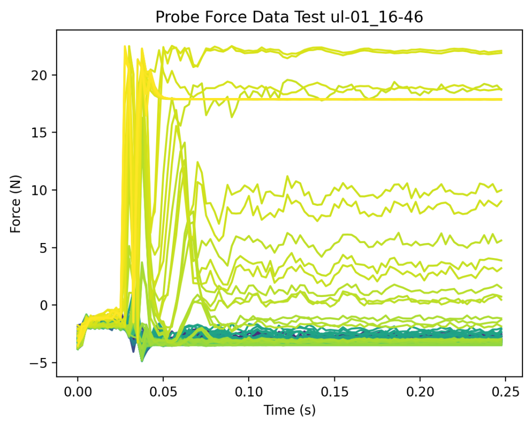

Fig. 12: Force sensor data from a typical needle insertion.

way to distinguish the two forces within a single probe event. However, looking at the trend as the needle is inserted aids in distinguishing the two. Based on our experience, we expect an increase in probe force when the needle encounters stiff tissue such as ligaments or connective tissue within the muscle or near the bone. Meanwhile, we would expect that frictional force due to a bending needle would gradually increase with insertion depth as the needle bends.

Our force data generally show patterns that match the force coming from changes in tissue composition rather than needle bending. The force data in most of our tests look similar to Figure 12 where the steady-state force readings are relatively independent of depth until the last few probe depths before bone is struck. The increase in force in relation to depth is sudden rather than gradual. In the other tests, such as the that shown in Figure 10, there are more intermediate steady-state force readings, indicating a more gradual force buildup. Looking carefully at those tests, though, it can be seen that the force buildup is not monotonic and will at times decrease as the needle gets deeper. This generally indicates that the needle is passing through a ligament or tissue boundary. This can be seen in Figure 10 as blue traces with increased force followed by deeper green traces with less force. Figure 11 shows that the needle passed through a layer of fatty tissue that likely contained stiff ligaments or other connective tissue at its boundary, thus causing the increased force readings of the blue traces followed by decreased readings of the green traces as the needle pierces it. The non-monotonic nature of the gradual-force-buildup needle insertions are inconsistent with the gradual buildup that we would expect from needle friction. The tests where the steady state force rose suddenly rather than gradually are also inconsistent with what we would expect from friction. Therefore our force data do not indicate significant amounts of friction due to needle bending.

Appendix III
Bone Strike Determination

Determining the needle depth when bone is struck is an important, though subtly difficult part of our data collection process. Manually verifying that the bone has been struck via dissection with the needle still in place is useful, but relying on that alone is not ideal. Firstly, on the surface of hard compact bone lies a relatively softer but still fibrous membrane called the periosteum. This can lead to ambiguity in determining where the bone starts and when the needle first struck it when observations of physical dissection alone are used. Based on the authors' experience, this ambiguity could be as high as 0.5–1 mm, which is fairly significant for our classification depth of 5 mm.

Secondly, once the tissue is dissected, the test must be stopped since the physical structure around the needle has been significantly altered. Individual tests are quite time-consuming, so the test operator must be reasonably sure that bone has been struck before stopping the test. It is possible to observe when the needle starts encountering significant resistance and use this as evidence that the needle has struck bone, but this approach is prone to subjectivity. Our testbed is not set up to electronically measure when the needle meets resistance during its advance, so test operators must rely on other observations, such as the sound of the stepper motor straining or a visible bowing of the needle. Such observations are not auditable like electronically recorded data are, making them more prone to human error. Also, failing to stop the needle soon enough could cause damage to the testbed, which could bias the operator's judgement.

To overcome this, we developed a technique for determining bone strike that relies on our electronically recorded data. It uses the fact that when the probe is fully extended, it extends beyond the needle by approximately 2 mm. During most of the needle insertion, the probe fully extends, with the probe motion ending due to the hard stop on the solenoid plunger, leading to a consistent reading on the distance data. When the probe gets within 2 mm of bone, the probe motion stops when the probe hits bone. As the needle gets closer, the probe hits sooner, which can be seen in the distance data. Finally, when the needle hits bone, any further advancement causes the bone to move with the needle; therefore, the distance that the probe extends stops decreasing. By looking for this pattern of decreasing probe extension followed by constant probe extension, the bone strike depth can be determined to be the depth at which the probe distance stopped decreasing.

It should be noted that when the needle strikes bone, advancing the needle further will only move the bone for a very small distance. If the needle is advanced too far, the needle will begin to bend and eventually—we theorize—catastrophically fail. Thus the pattern of the bone advancing with the needle only holds true for a short distance, but this small window is sufficient to utilize the previously described pattern to determine when bone strike has occurred.

Figure 13 shows this pattern occurring during a needle insertion. For clarity, most of the probe events that are not close to bone have been omitted. We classified the bone strike depth for this needle insertion as 41.5 mm. It can be seen that the probe motion readings are consistent for probe events far away from bone, with little difference between probes taken at 26 mm and at 38.5 mm. As the probe gets within about 2 mm of bone, the total distance travelled by the probe begins decreasing by approximately the same amount that the needle is advancing. Finally, when the probe hits bone, the probe

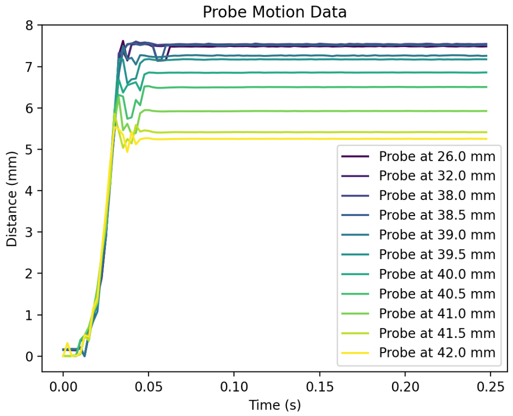

Fig. 13: Probe motion data plotted at depths far from bone and at depths immediately preceding bone strike. For this test, bone strike occurred at a depth of 41.5 mm. The decreasing throw of the probe can be seen as it starts hitting bone with the throw becoming constant again once the needle strikes bone.

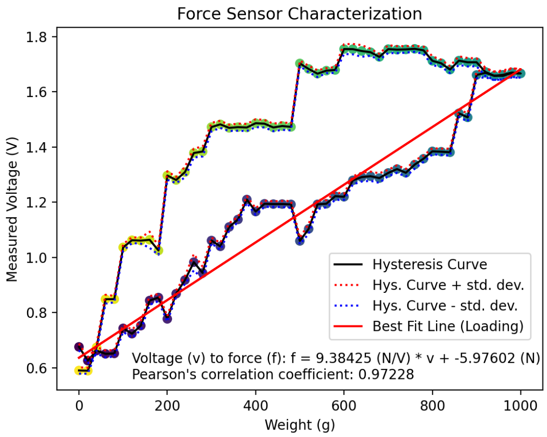

Fig. 14: Hysteresis curve for force sensor calibration. The loading curve starts with the purple point at 0 g and continues until the maximum weight at 1000 g. The unloading curve then follows from the maximum weight in brighter colors until it ends at the yellow point at 0 g.

distance stops decreasing as is seen in the probe events at 41.5 and 42 mm. Thus we measure the bone strike depth as the shallowest depth where the probe distance does not decrease, which is 41.5 mm in this case. This same pattern can be seen in other graphs of our distance data such as Figure 9, though the large number of probe events on that graph make it more difficult to see.

Appendix IV
Force Sensor Calibration

Figure 14 shows the calibration results for our force sensor. To calibrate the sensor, we made a pulley-like apparatus so that the gravitational force from calibration weights placed in a lightweight suspended container is transferred to a flat plate that moves horizontally. This plate was then placed against the extended probe of our needle apparatus, thus allowing us to apply force to the force sensor in the same orientation it is in during testing.

The calibration procedure began with no weights in the suspended container. Weights were added in 20 g increments until 1000 g was reached, after which weights were removed in 20 g decrements until the container was again unloaded. The points on the graph correspond to these specific weights. At each weight, the force on the probe was captured three times, with 100 force measurements taken per capture, and the average and standard deviations of each capture was taken. The averages and standard deviations of all three captures were both separately averaged in order to get the values shown in the graph. The average of the averages of the three captures corresponds to the voltage value of the point shown on the graph for that weight. The average of the three standard deviations for each point is shown by the distance between the point and the red and blue dotted lines above and below it. The fact that these red and blue lines stay close to the hysteresis curve shows that our force sensor has little variation.

A line of best fit was calculated using linear regression on the loading curve in order to obtain a transform from measured voltage to force. It has a Pearson correlation coefficient of 0.97 with the points in the loading curve, indicating a good fit. Only the points from the loading curve were used for two reasons. First, the loading curve is more reliable than the unloading curve. Friction between the flat plate and its guide led to inaccurate data on the unloading curve, which can be seen in its stairstep-like nature. Despite weight being removed, the plate would stick in the same location, exerting roughly the same amount of force until suddenly dislodging. This was less of an issue in the loading curve where the lightweight plate was increasingly being pulled in the direction of the applied force. Second, our force measurements are only taken when the probe is extending, which means that the force sensor is being loaded rather than unloaded (except for some small segments of unloading during bouncing).

All of the measurements shown in Figure 14 were taken with the probe extended. There were some measurements (which are not shown) taken with the probe retracted, some of which had a different force reading than the 0 g point. One was approximately 0.2 V or 1.9 N lower than the purple 0 g point in Figure 14. This could indicate a position-dependent measurement error in our force sensing setup. While not ideal, such an error would account for less than 10% of the sensor's dynamic range, which, as seen in graphs of our force data, is small compared to the changes in force data as the needle approaches bone. Furthermore, this error affects all of our probe data, which means that the machine learning classifiers will adapt to it since they are looking for differences between probe measurements rather than comparisons of probe measurements to ideally calibrated numerical force values.

Appendix V
Silicone Results

This section adds further detail about preliminary testing on silicone of different hardnesses described in Section IV-A. One of these silicone samples was prepared to simulate ligament tissue, which we expect an epidural needle to enter before

hitting bone or close to the epidural space, while the other one was prepared to simulate adipose tissue, which we expect to encounter closer to the skin. We expected ligaments to have a hardness of 20A (hardness of 20 on a shore A scale) and thus used Dragon Skin 20 (Smooth-On; Macungie, PA, USA), which is designed to have that hardness. To approximate adipose tissue, we used the silicone additive Slacker (also made by Smooth-On) to soften the Dragon Skin 20. Our mix ratio was 1 part Dragon Skin A : 1 part Dragon Skin B : 2 parts Slacker. Note that Dragon Skin 20 comes in two parts that are mixed in a 1:1 ratio.

We did not have equipment for measuring the durometer value of the two silicones. According to its data sheet, the Dragon Skin 20—and thus our simulated ligament tissue—has a hardness of 20A. Smooth-On's data sheets do not give a durometer value for the specific mix we used for our adipose tissue. Smooth-On does provide durometer values for mixes that use the same mix ratio (1A:1B:2S) but use softer versions of Dragon Skin which are designed to have durometer values of 10A when no Slacker is used. The durometer values for these mixtures range from 000-7 to 000-51, which can serve as a lower limit for the durometer value of our simulated adipose tissue. An anesthesiologist on our team noted that our silicone samples feel like ligament and adipose tissues.

Figure 15 shows force, distance, force FFT, and distance FFT readings for both silicones. These readings were taken with an early version of the prototype, which used a force sensor with less precision than our current sensor and for which we never calculated an exact voltage-to-force conversion. Therefore, the force units are reported in volts. Also, due to the nature of our earlier measurement circuitry, lower voltages correspond to higher forces.

Despite the lower-precision force sensor, the differences between the two types of silicone are readily apparent, especially in the force graphs. The difference can also be seen in the approximated viscoelastic response graphs shown in Figure 16, which were generated by dividing the force FFT values by the distance FFT values. There is a clear separation of the two types of silicone in the range of 0–25 Hz. It is unclear whether the responses above 25 Hz are due to lack of material differences at those frequencies or limitations of the measurement capabilities of our early testbed.

Appendix VI
Sensor details

Force Sensor

- Manufacturer: FUTEK
- Manufacturer location: Irvine, CA, USA
- Model: LCM100
- Item #: FSH03829 (5 lb capacity version)
- Resolution: 2.9×10^{-2} N

Distance Sensor

- Manufacturer: iC Haus
- Manufacturer location: Bodenheim, Germany
- Model: iC-MU
- Resolution: 3.1×10^{-4} mm

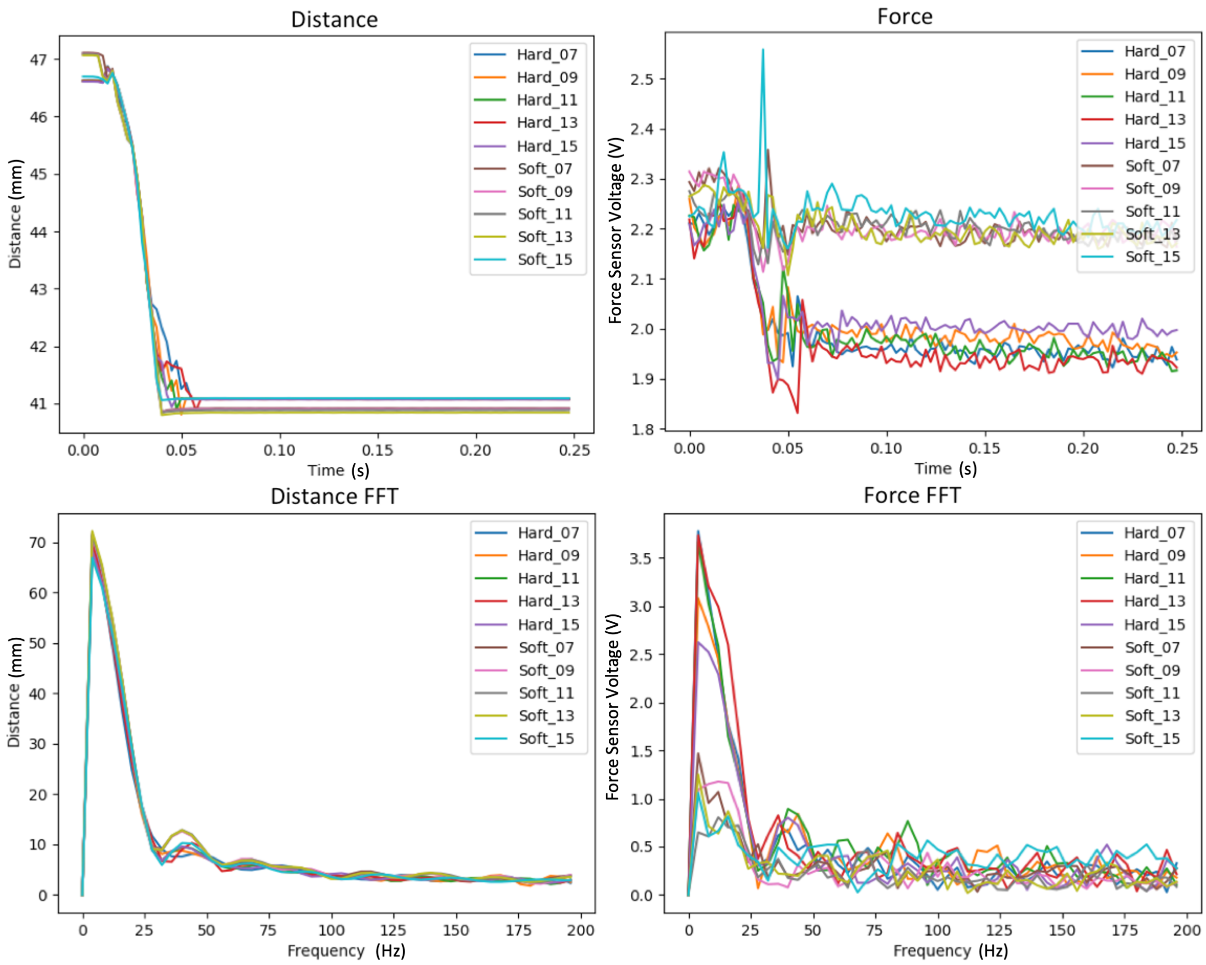

Fig. 15: Graph of raw force and distance data and their FFTs collected from test silicones. Each line is one probe. "Hard" indicates the ligament-like silicone. "Soft" indicates the adipose-like silicone.

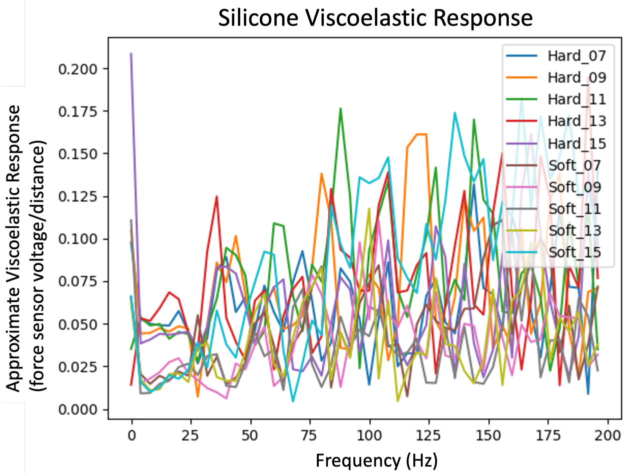

Fig. 16: Graph of approximated viscoelastic responses of our test silicones. Each line is one probe. "Hard" indicates the ligament-like silicone. "Soft" indicates the adipose-like silicone.

Appendix VII
Feature Descriptions

This section describes the features that we analyzed for use in our classification algorithm as well as the makeup of the feature sets used.

A. Individual Feature Analysis

In order to evaluate the suitability of different features for classification, we generated graphs of each feature from our training data, allowing for visual inspection. Figures 17–68 show the resulting graphs. There are two types of graph: a line plot for scalar features (such as an average or a standard deviation) and heatmap plots for vector features (such as the raw data vectors or FFTs). In each graph, the x-axis is the distance from bone or "depth class", which decreases from left to right until it hits 0 mm, indicating a bone strike. On the scalar graphs, the y-axis is the value of the given measurement. On the heatmaps, the y-axis corresponds to individual vector elements where the value of each element is indicated by the cell's color. The vector elements are in increasing order from top to bottom. In the raw data heatmaps, this means that the y-axis is time while in the FFT heatmaps, the y-axis is frequency bin.

Both types of graphs show the mean of their specific statistic across all needle insertions in the training set. This means each heatmap shows the mean of the individual heatmaps for each needle insertion. The line graphs show the mean as a blue line. Additionally, each line graph shows the plot for each individual needle insertion in the training set as gray lines. The heatmaps do not contain any visual representation of individual needle insertions.

On each graph, there is a vertical red line indicating a depth of 5 mm from bone. This was used to help determine which features start providing information before the needle is within 5 mm of bone. The results of this analysis were used to aid in generating the feature sets described in Section VII-B.

Note that the needle insertions on which the graphs are based do not all have the same depth; therefore, not all needle depth data points exist in all graphs. The insertions in the graphs below have been aligned to place bone strikes at the same depth. Since some of the insertions are longer than others, the higher depth classes (those far from bone) may only contain data from a subset of all the needle insertions in the graph. This can lead to artifacts that appear as sudden changes in value when a depth class is reached where a new needle insertion begins due to averaging. Those sudden changes do not reflect sudden changes within any individual needle insertion but rather reflect that the set of needle insertions being averaged has changed. The gray lines in the scalar feature graphs reveal the depth classes at which individual needle insertions begin.

The title of each graph describes the feature. Some are self-explanatory such as the raw force data (Figure 17), the raw distance data (Figure 18), the FFT of the force data (Figure 19), and the FFT of the distance data (Figure 20). The rest represent data extracted from subsequences and have a naming scheme of "seq=X_Y_Z".

X indicates the subsequence. The time ranges of the individual subsequences are shown and described in Figure 5 and its caption. The meanings of the subsequence labels are as follows:

- ic: initial conditions,
- mv: step input (probe movement),
- fu: unsteady force (force unsteady),
- ss: steady state, and
- all: all data points in the sequence.

Y indicates the type of data. "force data" [volts] is the raw force sensor data in volts. The voltage values are linearly related to force values and can be converted to newtons using the equation in Appendix IV. "dist data" [mm] is the probe position data. The distance data values on the graph correspond to the absolute position measurements on our encoder and therefore are offset from zero. Other distance graphs in this paper normalize the raw measurements by subtracting each data point from the value of the first point.

Z indicates the statistic extracted from the subsequence among these as follows:

- fft: an FFT of the data points in the subsequence,
- mean: the mean of the data points in the subsequence,
- std dev: the standard deviation of the data points in the subsequence,
- skew: the skewness of the data points in the subsequence, and
- kurtosis: the kurtosis of the data points in the subsequence.

B. Feature Groups

Table II shows the features in each feature group.

TABLE II: Features Used in Each of Our Feature Groups

Feature	Force FFT ∪ Dist FFT	Force ∪ Dist	Raw ∪ Stats	Subsequence Force FFTs
dist data		X	X	
force data		X	X	
fft-dist data	X			
fft-force data	X			
seq=all_dist data_kurtosis				
seq=all_dist data_mean			X	
seq=all_dist data_skew				
seq=all_dist data_std dev			X	
seq=all_force data_kurtosis				
seq=all_force data_mean			X	
seq=all_force data_skew				
seq=all_force data_std dev			X	
seq=fu_dist data_fft				
seq=fu_dist data_kurtosis				
seq=fu_dist data_mean				
seq=fu_dist data_skew				
seq=fu_dist data_std dev				
seq=fu_force data_fft				X
seq=fu_force data_kurtosis				
seq=fu_force data_mean			X	
seq=fu_force data_skew				
seq=fu_force data_std dev			X	
seq=ic_dist data_fft				
seq=ic_dist data_kurtosis				
seq=ic_dist data_mean			X	
seq=ic_dist data_skew				
seq=ic_dist data_std dev			X	
seq=ic_force data_fft				X
seq=ic_force data_kurtosis				
seq=ic_force data_mean			X	
seq=ic_force data_skew				
seq=ic_force data_std dev			X	
seq=mv_dist data_fft				
seq=mv_dist data_kurtosis				
seq=mv_dist data_mean				
seq=mv_dist data_skew				
seq=mv_dist data_std dev				
seq=mv_force data_fft				X
seq=mv_force data_kurtosis				
seq=mv_force data_mean				
seq=mv_force data_skew				
seq=mv_force data_std dev				
seq=ss_dist data_fft				
seq=ss_dist data_kurtosis				
seq=ss_dist data_mean			X	
seq=ss_dist data_skew				
seq=ss_dist data_std dev			X	
seq=ss_force data_fft				X
seq=ss_force data_kurtosis				
seq=ss_force data_mean			X	
seq=ss_force data_skew				
seq=ss_force data_std dev			X	

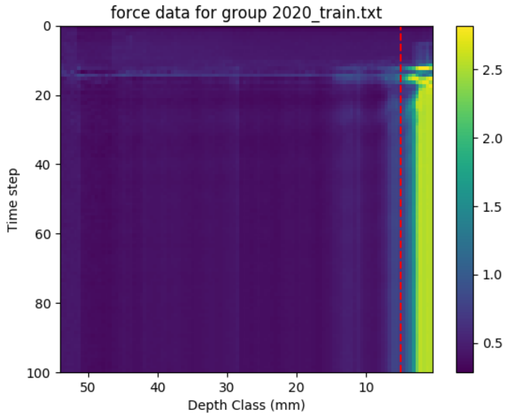

Fig. 17

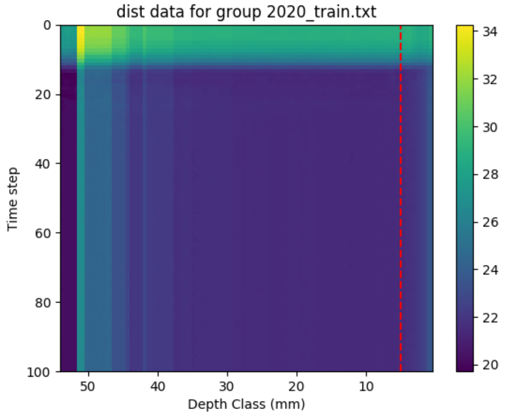

Fig. 18

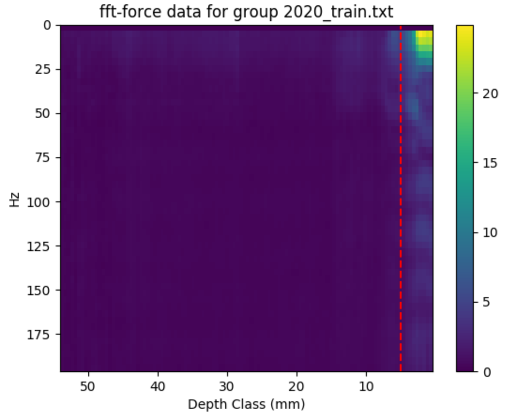

Fig. 19

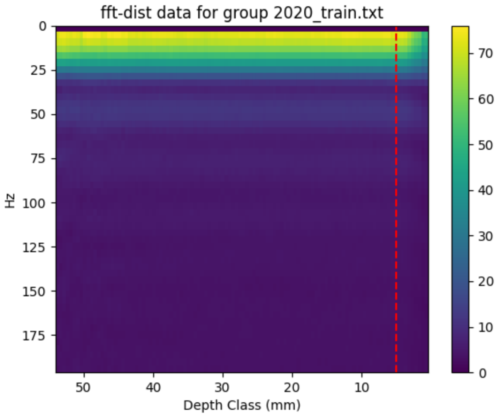

Fig. 20

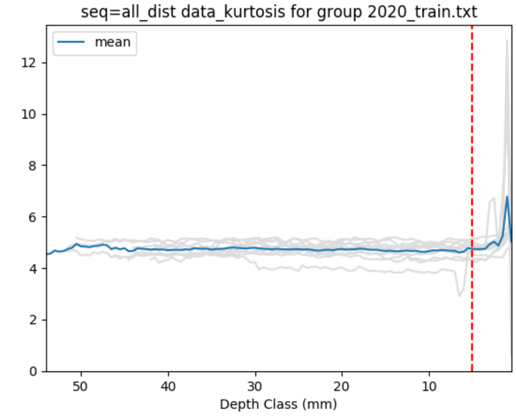

Fig. 21

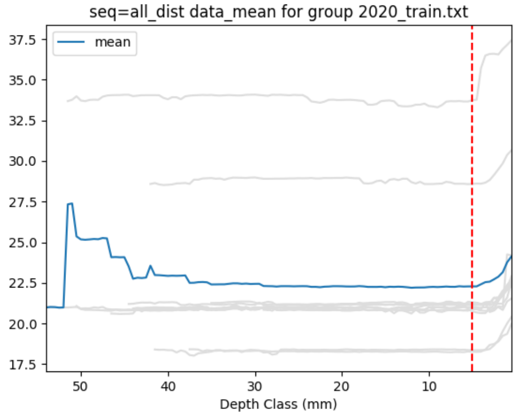

Fig. 22

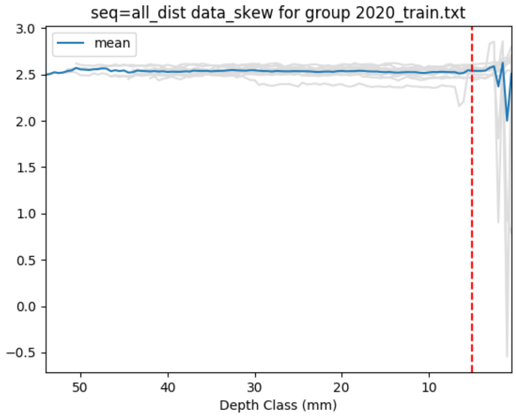

Fig. 23

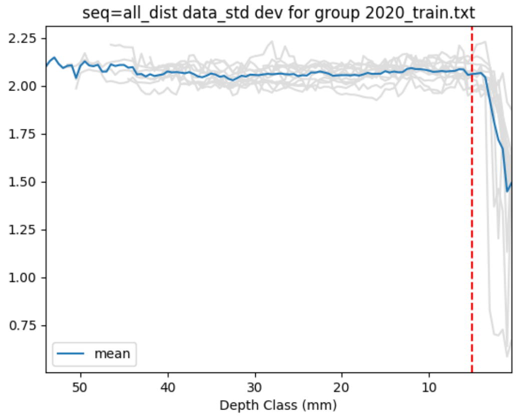

Fig. 24

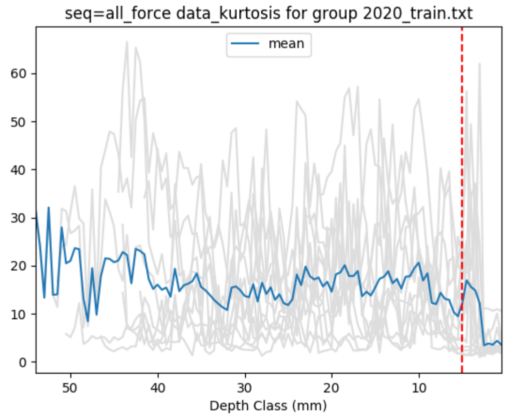

Fig. 25

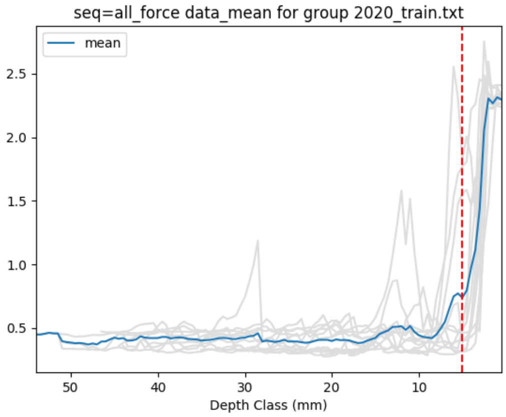

Fig. 26

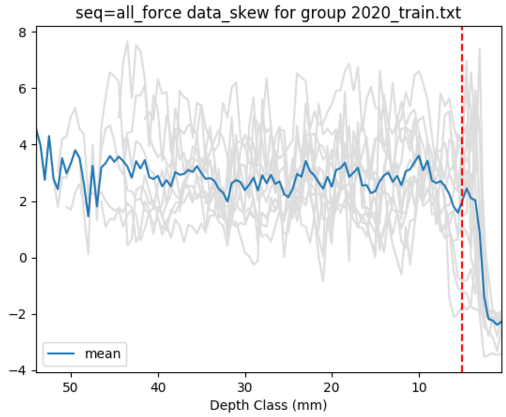

Fig. 27

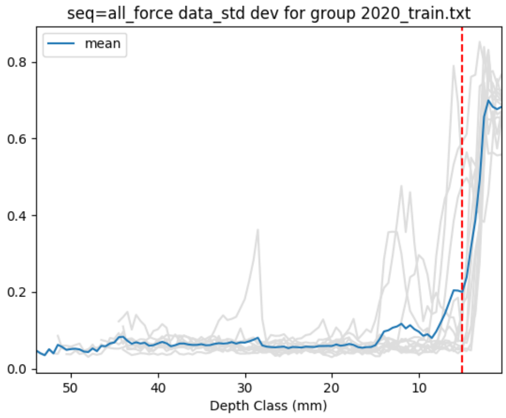

Fig. 28

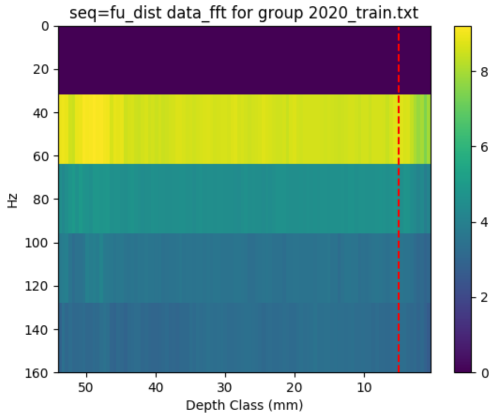

Fig. 29

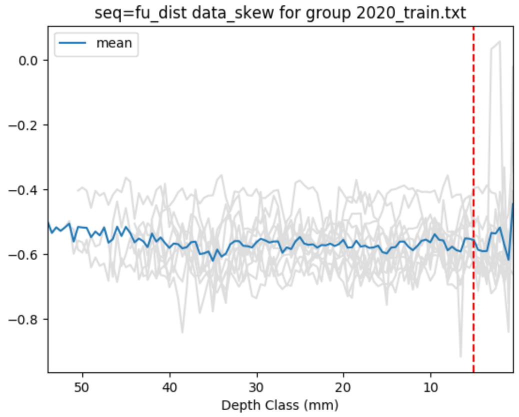

Fig. 32

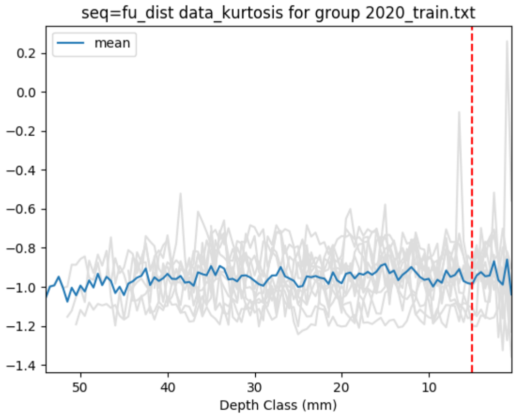

Fig. 30

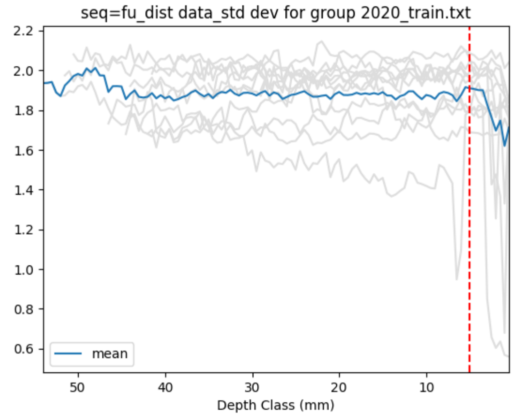

Fig. 33

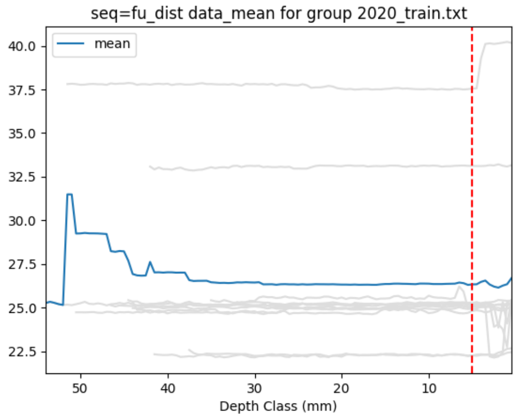

Fig. 31

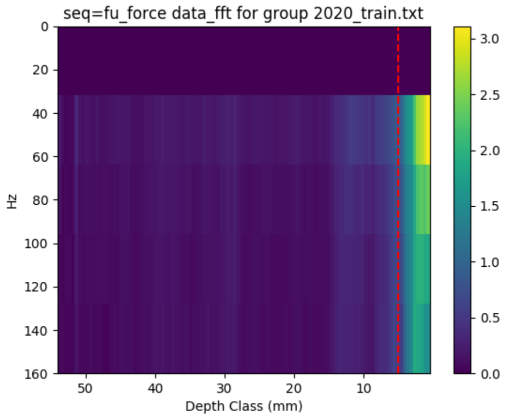

Fig. 34

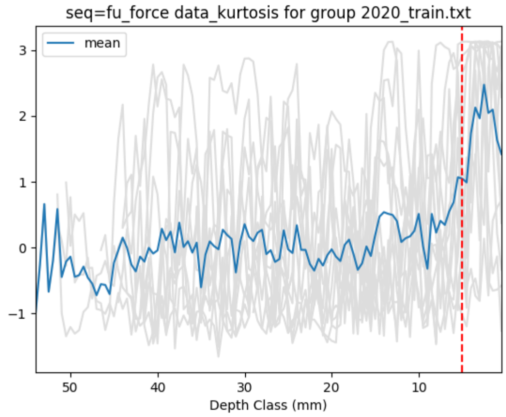

Fig. 35

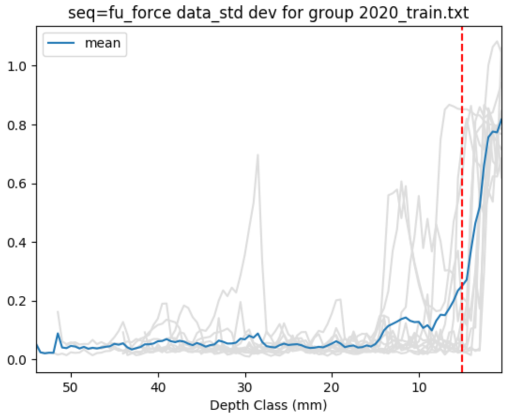

Fig. 38

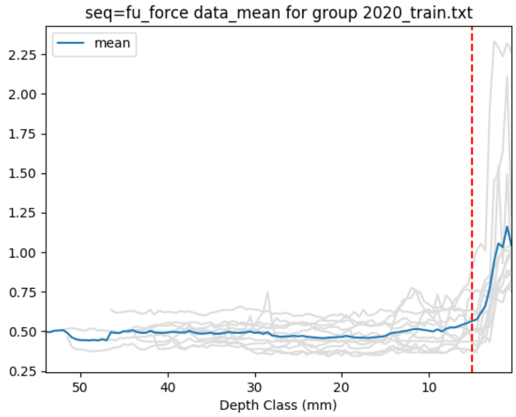

Fig. 36

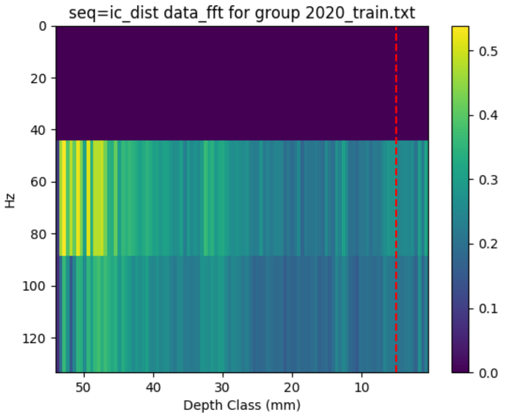

Fig. 39

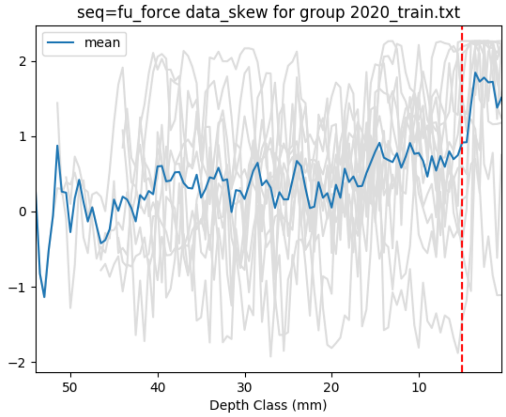

Fig. 37

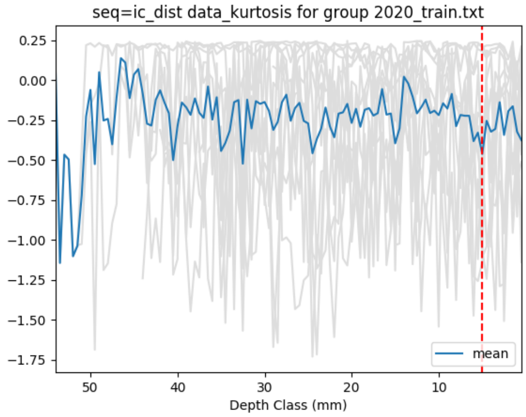

Fig. 40

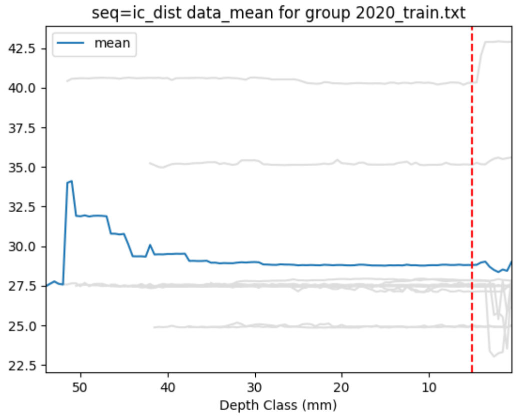

Fig. 41

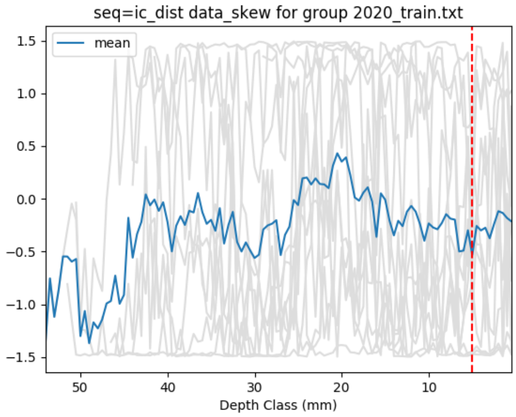

Fig. 42

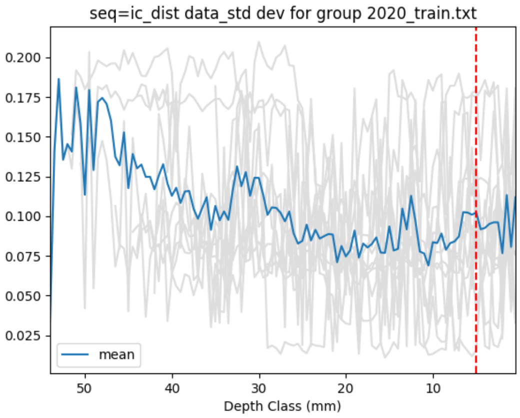

Fig. 43

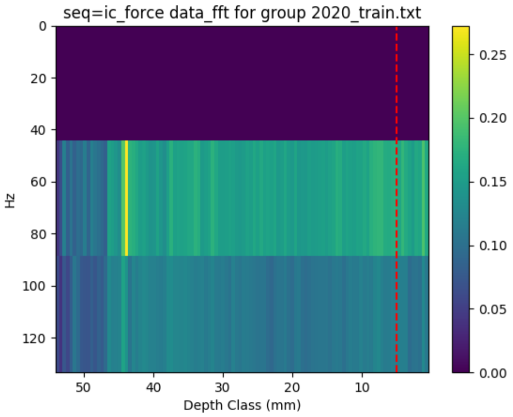

Fig. 44

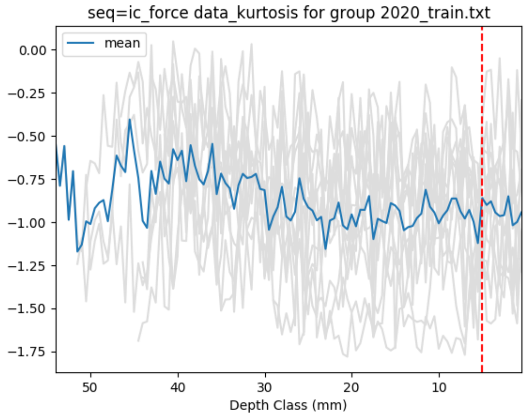

Fig. 45

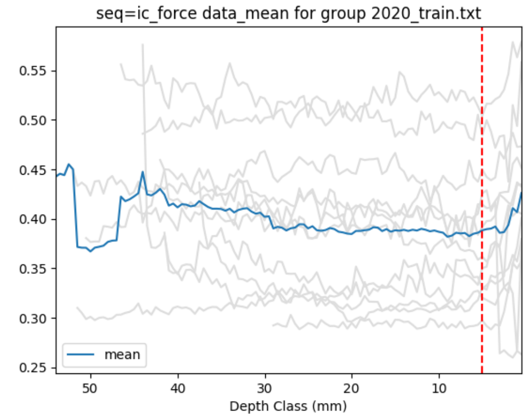

Fig. 46

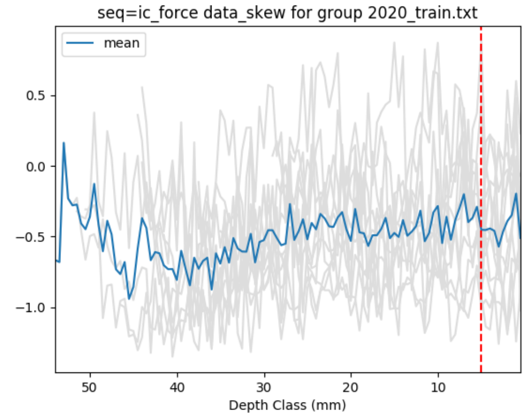

Fig. 47

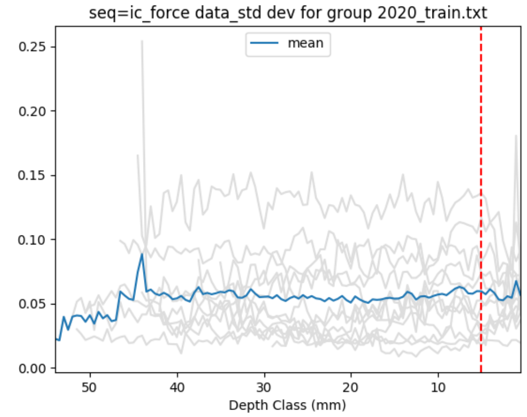

Fig. 48

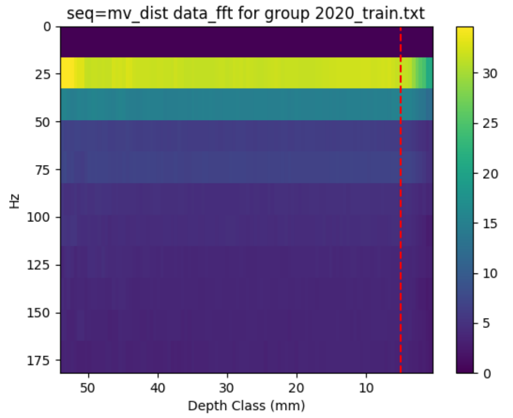

Fig. 49

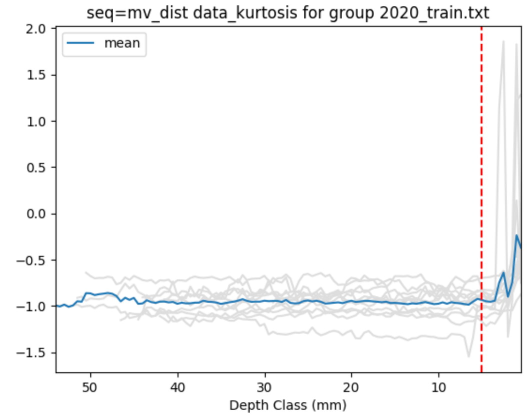

Fig. 50

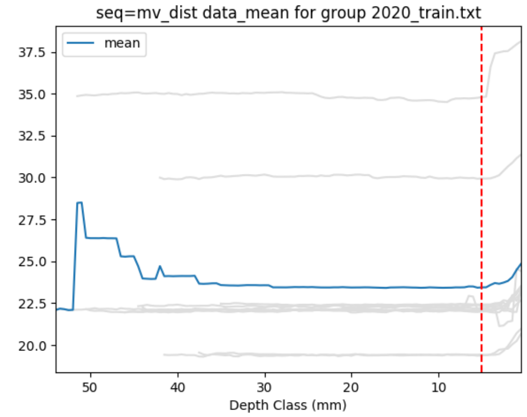

Fig. 51

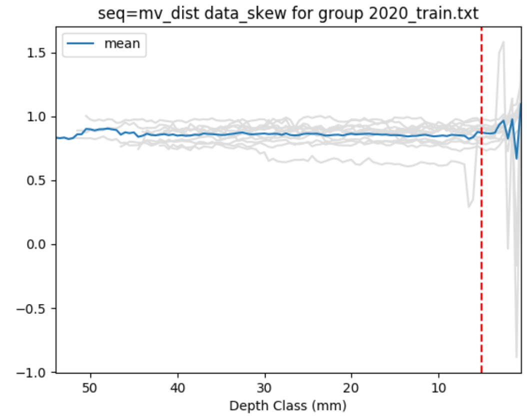

Fig. 52

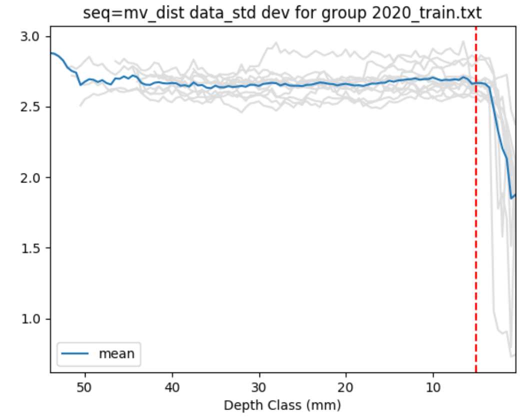

Fig. 53

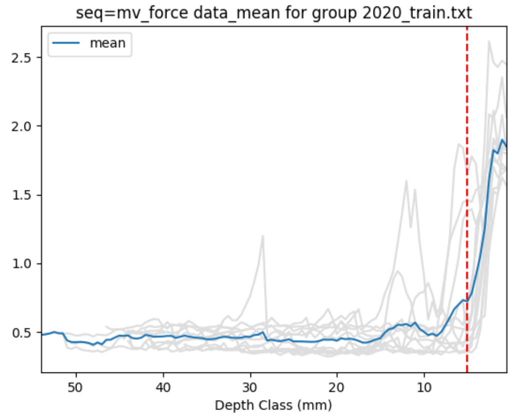

Fig. 56

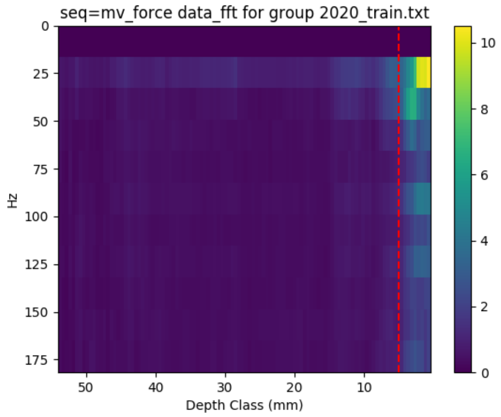

Fig. 54

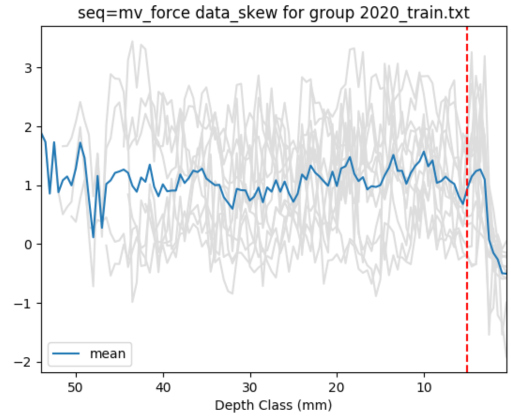

Fig. 57

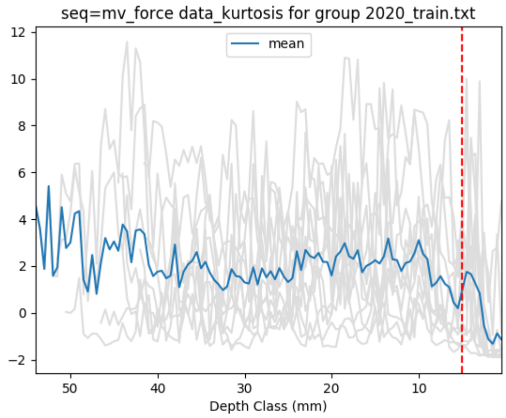

Fig. 55

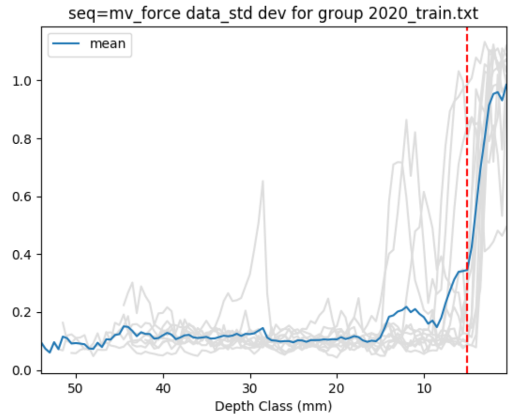

Fig. 58

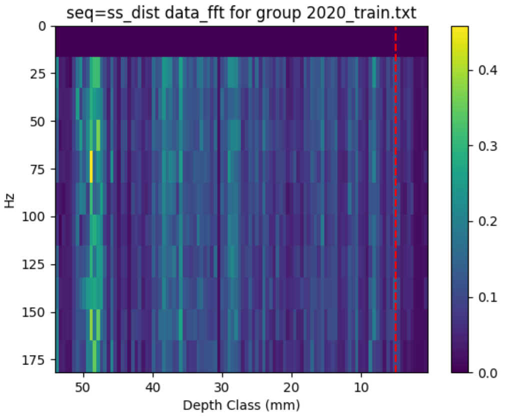

Fig. 59

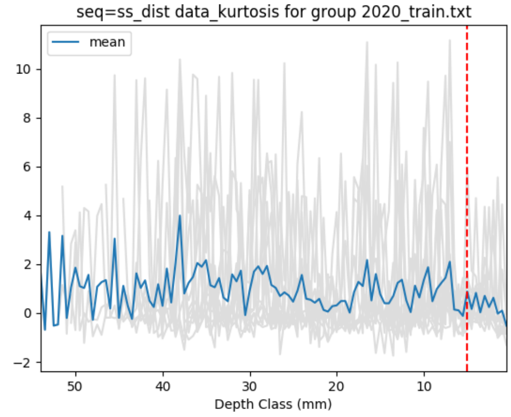

Fig. 60

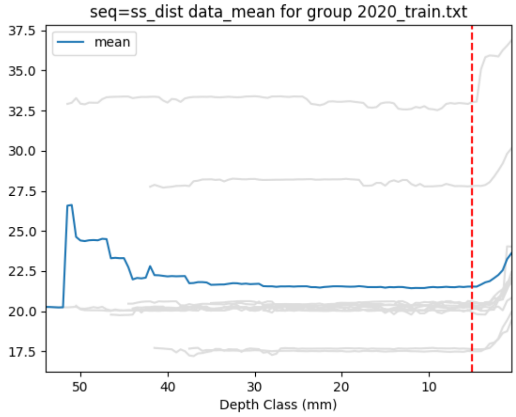

Fig. 61

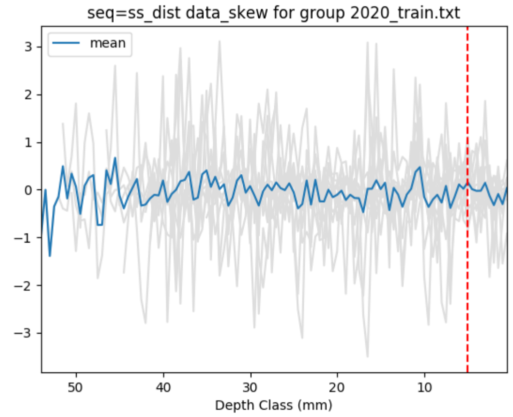

Fig. 62

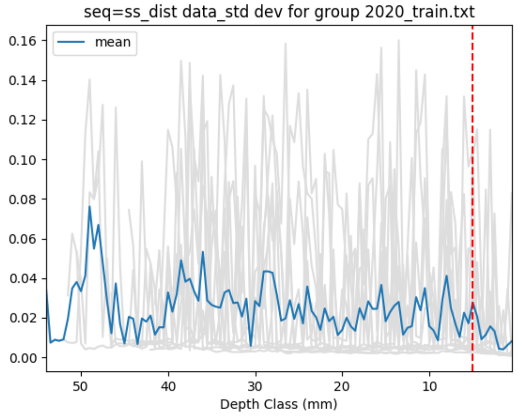

Fig. 63

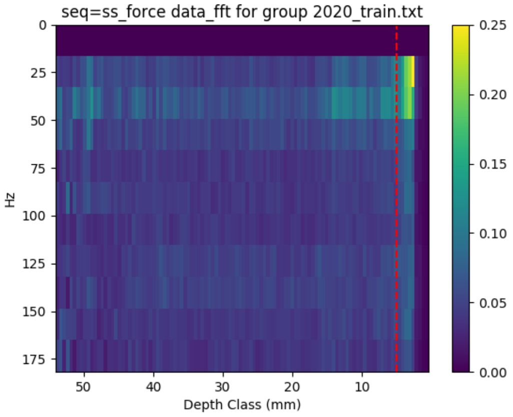

Fig. 64

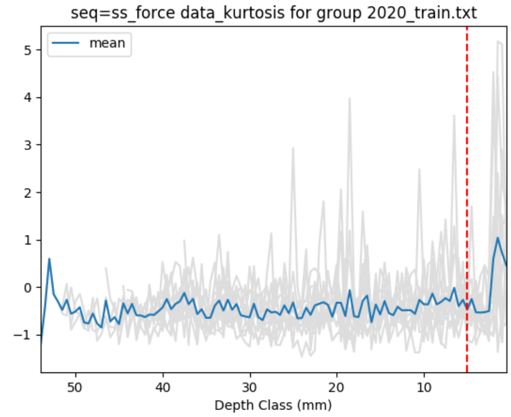

Fig. 65

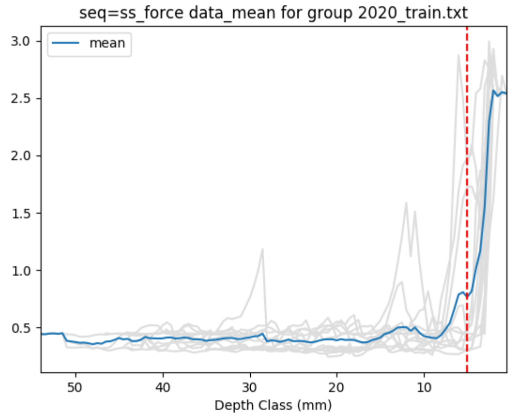

Fig. 66

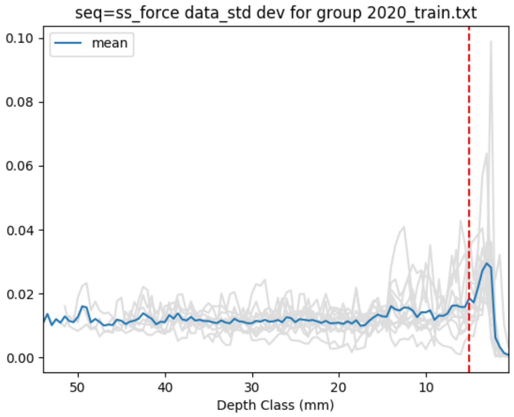

Fig. 68

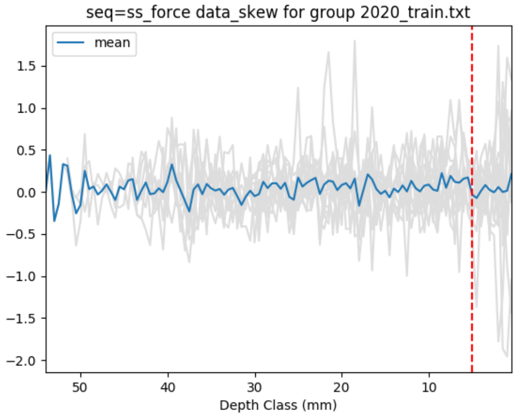

Fig. 67
